# Statistical performance indicators and index—a new tool to measure country statistical capacity

**DOI:** 10.1038/s41597-023-01971-0

**Published:** 2023-03-20

**Authors:** Hai-Anh H. Dang, John Pullinger, Umar Serajuddin, Brian Stacy

**Affiliations:** 1 World Bank, 1818 H Street NW, Washington, DC 20433 USA; 2 International School, Vietnam National University, Hanoi, 144 Xuan Thuy, Cau Giay, Hanoi, Vietnam; 3 Indiana University, Bloomington, 107 S. Indiana Avenue, Bloomington, IN 47405 USA; 4 IZA, Schaumburg-Lippe-Straße 5–9, Bonn, 53113 Germany; 5 United Kingdom National Statistical Service, 2 Marsham Street, London, SW1P 4DF UK

**Keywords:** Economics, Developing world, Interdisciplinary studies

## Abstract

Strong statistical capacity is a prerequisite for producing reliable statistics that helps monitor a country’s governance and economic performance. This is particularly relevant for a large number of poorer countries, which have weaker statistical capacity but have to rely more on these statistics for various objectives such as monitoring poverty reduction or reporting to international donors. We present the Statistical Performance Indicators and Index (SPI) as the World Bank’s new official tool to measure country statistical capacity. The SPI is conceptually motivated, builds on a mathematical foundation, and significantly expands the number of indicators and the number of covered countries compared to its predecessor. The new index has a strong correlation with other common development indicators such as GDP per capita, governance, human capital, poverty, and inequality. It can also accommodate future improvements as the global data landscape evolves.

## Background & Summary

National statistical systems (NSSs) face the challenge to adapt and develop in order to meet the rapidly evolving needs of various data users. The key task for the NSS is to provide accurate and updated data such that stakeholders, including policy makers and the general public, can make well-informed policy decisions. Another recent and no less important task for the NSS is to monitor progress on the Sustainable Development Goals (SDGs), which requires high-quality and comparable data across countries. Indeed, without reliable data, a one-percentage-point decrease in the poverty rate—or a similar increase in the school enrolment rate—in one country might not be comparable to the corresponding figure in another.

Examples abound that abrupt changes to key indicators can lead to major policy revisions for a country and subsequently have consequential effects on its economy. For instance, after Ghana updated its national accounts methodology, this country’s Gross Domestic Product (GDP) increased by 60 percent, which in turn led to a lower debt-to-GDP ratio and reclassification of the country status from a low-income economy to a middle-income economy (Devarajan, 2013^1^; Jerven, 2013^2^). Revisions to the base year for GDP estimation raised Nigeria’s GDP even more by 89 percent (Economist, 2014)^3^. In addition to impacts on the countries’ own macro-economic policies, these changes would likely have influenced their access to preferential loans and foreign aid through international organizations. Accordingly, the international community has placed much attention on assessing and improving country statistical capacity, particularly for (poorer) countries with weaker capacity.

The Statistical Capacity Indicators and Index (SCI) is a tool developed by the World Bank in 2004 to assess the effectiveness of its lending projects related to improvements in countries’ statistical capacity (World Bank, 2020a)^4^. The SCI was also widely employed by different international and national agencies for various monitoring objectives (United Nations, 2016^5^; OIC, 2012^6^; UNICEF, 2018^7^) and in academic research on improving statistical capacity and its relationship with country governance and economic growth (Sanga *et al*., 2011^8^; Beegle *et al.,* 2016^9^; Tapsoba *et al*., 2017^10^; Hoogeveen and Nguyen, 2019^11^; Arezki *et al*., 2020^12^; Angrist *et al*., 2021^13^). Comparing the SCI with the statistical capacity measurement indexes used by other organizations, including the Partnership in Statistics for Development in the 21^st^ Century (PARIS21), the Food and Agriculture Organization of the United Nations (FAO), the United Nations Economic Commission for Europe (UNECE), the United Nations Economic Commission for Africa (UNECA), and the U.S. Census Bureau, Dang *et al*., 2021^14^ observe that the SCI is the only tool that provides comparable data across different countries over time. The SCI also covers the largest number of countries (i.e., 146 countries) over the longest period (i.e., from 2004 to 2020).

Yet, the SCI has several key limitations. Despite recent advances with data computing and storage capacity as well as more stringent data requests (e.g., for the Sustainable Development Goals (SDGs)), the SCI’s methodology and coverage have remained the same since its inception. The SCI’s focus on poorer countries also limits its relevance for global analysis. Finally, key concepts underlying data production and data usage are unavailable, further contributing to the misconceptions that all the stakeholders use similar standards in safe-guarding data quality and make similar efforts to ensure open data access. Moreover, the index’s aggregation method lacks a solid foundation (Ngaruko, 2008)^15^.

These concerns have practical relevance. Two notable examples stand out. First, the SCI includes no indicators for labor force surveys and establishment surveys, which are indispensable instruments in a modern NSS’s toolbox to monitor the latest (un)employment trends in the economy. Second, the overall SCI scores of Cameroon and Sudan rose by around 24 percent in one year, between 2015 and 2016. This stands in sharp contrast with the common understanding that a country’s statistical capacity often improves incrementally.

We present a new set of statistical indicators and index that respond to the modern global data landscape. Conceptually, we identify five key pillars regarding data usage and production in any modern economies—data use, data services, data products, data sources, and data infrastructure—to examine a country’s statistical performance. We subsequently construct an updated set of Statistical Performance Indicators and a new index based on these indicators, hereafter referred to as the Statistical Performance Index (SPI).

Empirically, the SPI covers 181 (low-, medium-, and high-income) countries and 51 indicators. Most importantly, it distinguishes all the countries’ statistical performance with a unique score. (This compares favourably with the SCI, which covers 145 countries—excluding most high-income countries—has fewer than 50 country unique scores and only 25 indicators). The SPI officially replaces the SCI as the World Bank’s main tool to measure country statistical performance, and it is open-data and open-code where users can freely access data and experiment with different adjustments to the index on the World Bank’s website. It has been adopted in various policy reports and publications.

A quick introduction to the SPI in terms of commonly asked questions and their answers and how to contact the World Bank’s SPI team is provided on the SPI home page (https://www.worldbank.org/en/programs/statistical-performance-indicators/about-spi#4). In addition, various blog posts and summary pieces about the SPI, with full references to more technical details, are provided on the following link https://www.worldbank.org/en/programs/statistical-performance-indicators/Blogs.

## Methods

### Conceptual motivations

While measuring a country’s statistical capacity is our ultimate goal, this task is difficult, if not impossible to implement at scale for all countries, given the typically unobserved inherent characteristics with an NSS. It is, however, relatively more straightforward to measure a country’s statistical performance through objective and comparable indicators. This challenge is highlighted by a large number of indicators with missing data that we discuss later.

An NSS can be characterized in a similar way to other organizational systems: beneficial outcomes for stakeholders are delivered through organizational outputs (services delivered) arising from effective internal processes that draw on a variety of inputs. The whole system can flourish if there is a strong infrastructure to support it. In recent years, UNECE and the Statistical Office of the European Communities (Eurostat) have done a considerable amount of work to conceptualize the statistical value chain to help modernize statistical systems. The Generic Statistical Business Process Model (GSBPM) has been used by many countries to guide development of their NSS alongside the Generic Activity Model for Statistical Organizations (GAMSO). In the European Union, the model has been developed into a detailed handbook (UNECE, 2019)^16^.

In order to understand how to make these generic models operational, PARIS21, Open Data Watch (ODW), the Global Partnership for Sustainable Development Data, the Organization for Economic Cooperation and Development (OECD) and others have defined a virtuous data cycle where the end goals of data use, data value and data impact are achieved through building up each element of the data ecosystem. The approach used in the SPI draws on these underpinning concepts.

**Fig. 1:**
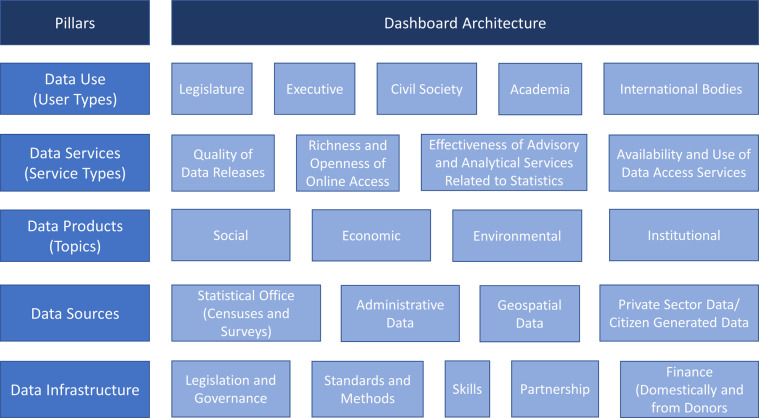
The Pillars and Dimensions that Construct the New SPI. The SPI is composed of 5 pillars and 22 dimensions mapped to each of the 5 pillars representing key areas of the statistical system.

We identify five key pillars of a country’s statistical performance, as shown in Fig. 1. These are data use, data services, data products, data sources, and data infrastructure, which can be further disaggregated into 22 dimensions. This figure shows these pillars and dimensions in the form of a dashboard, which can help countries identify areas for development in their statistical system. We briefly describe these pillars below and provide more details on the dimensions of the SPI in the in the Supplementary Information, Appendix A, Supplementary Table A.1.

Since statistics have no value unless they are used, the first pillar of the SPI is data use. In order to meet user needs, the statistical system must develop a range of services that connect data users and producers and facilitate dialogue between them. The second pillar of the SPI is therefore data services that are trusted by users. The dialogue between users and suppliers in turn drives the design of statistical products that are to be created including the quality of product needed for the country requirement. This will incorporate accuracy, timeliness, frequency, comparability, and levels of disaggregation. The third pillar of the SPI is therefore data products. In order to create the products required, the statistical system needs to make use of a variety of sources from both inside and outside the government. This includes making use of typical data collection methods like censuses and surveys, but also administrative data, geospatial data, and data generated from the private sector and from citizens. The fourth pillar of the SPI is therefore data sources. For the cycle to be complete, capability needs continuously to be reviewed to ensure that it is enough to deliver the products, services and ultimately data use required. The fifth pillar of the SPI is therefore data infrastructure. In summary, a successful statistical system offers highly valued and well-used statistical services, generates high quality statistical indicators that can also track progress for the SDGs, draws on all types of data sources relevant to the indicators that are to be produced, develops both hard infrastructure (legislation, governance, standards) and soft infrastructure (skills, partnerships), and has the financial resources to deliver.

Supplementary Figure A.1 in Appendix A offers an alternative visual description of the beneficial interactions of the different data pillars, which reinforce each other through stakeholders’ partnership, joint accountability, better capacity, and meeting user needs. Improvements in performance can be represented as a virtuous data cycle that can become self-sustaining.

### Construction of the SPI

We employ Cameron *et al*.’s (2021)^17^ nested weighting structure to construct the Statistical Performance Index (overall score). Compared to other weighting schemes, this weighting structure offers properties such as symmetry, monotonicity, and subgroup decomposability. It is based on Atkinson’s (2003)^18^ counting method, which was employed to construct a social exclusion index (Chakravarty and D’Ambrosio, 2006)^19^ and to measure adjusted multi-dimensional poverty (Alkire and Foster, 2011)^20^. Our statistical performance indicators have a three-level structure, and the SPI overall score is formed by sequentially aggregating the indicators at each level.

To begin we produce a score for each dimension within a given pillar, which, unless otherwise stated, is an unweighted average of the indicators within that dimension

1 $$SPI.DI{M}_{ctpd}={\sum }_{i=1}^{{N}_{I}}\frac{SPI.IN{D}_{ctpdi}}{{N}_{I}}$$

where *SPI.IND*_*ctpdi*_ is an indicator *i* in dimension *d*, pillar *p*, time period *t*, and country *c*, and *N*_*I*_ is the number of indicators in dimension *d*. For instance, the score for the Standards and Methods dimension is obtained by taking the unweighted average of all indicators in this dimension, including the indicators for the system of national accounts in use, national accounts base year, classification of national industry, Consumer Price Index (CPI) base year, and classification of household consumption (Appendix A, Supplementary Table A.1).

A score for each pillar is subsequently computed as the average score of the dimensions in that pillar. For pillars 1, 2, 4, and 5, the unweighted average of the dimensions within each pillar is taken. For pillar 3 on data products, we take a weighted average of the dimensions, where the weights are based on the number of SDGs in each dimension (6 SDGs in dimension 3.1 on social statistics, 6 SDGs in dimension 3.2 on economic statistics, 2 SDGs in dimension 3.3 on environmental statistics, and 2 SDGs in dimension 3.4 on institutional statistics). Note SDG 14 - Life Below Water - is omitted because land-locked countries do not report on these indicators.

This reflects a perspective that all SDGs are of equal importance, and therefore the dimensions are weighted accordingly. Additionally, for pillar 4 on data sources, censuses and surveys are given separate weights, so that censuses, surveys, administrative data, and geospatial data each receives a weight of 1/4. While censuses and surveys are in the same pillar in the framework, and therefore each would typically only receive a weight of 1/6 in this dimension, because of their importance in producing many indicators, they are given extra weight such that each gets a weight of 1/4.

The score for each pillar (*SPI.PIL*_*ctp*_) is calculated as follows

2 $$SPI.PI{L}_{ctp}={\sum }_{d=1}^{{N}_{d}}\frac{{\omega }_{pd}\times SPI.DI{M}_{ctpd}}{{N}_{d}}$$

*where ω*_*dp*_ is the weight for dimension *d* in pillar *p*, and *N*_*d*_ is the number of dimensions in pillar *p*.

Finally, the SPI overall score for country *c* in time *t* is derived by taking the average across the 5 pillars. The SPI overall score has a maximum score of 100 and a minimum of 0. A score of 100 would indicate that a country has every single element that we measure in place. A score of 0 indicates that none is in place. The SPI overall score (*SPI.INDEX*_*ct*_) is calculated as follows

3 $$SPI.INDE{X}_{ct}={\sum }_{p=1}^{{N}_{p}}\frac{SPI.PI{L}_{ctp}}{{N}_{p}}$$

where *SPI.PIL*_*ctp*_ is the SPI pillar scores for country *c* in time *t* for the five pillars discussed above, and *N*_*p*_ is the number of pillars. We provide more technical discussion on the SPI properties in Appendix B.

### Data collection

The SPI draws on a variety of data sources to create the indicators. A guiding principle is that the SPI relies on openly available data from credible sources, such as international organizations and national statistical office (NSO) websites. The SPI team used web scraping, accessed publicly available databases, or in some cases visited NSO websites to acquire the information. While greater detail for each specific indicator can be found in the technical documentation (Dang *et al*., 2021)^21^ describing each indicator, a general overview is provided here. Because much of the data collection process has been automated, updating the SPI with a preliminary dataset each year requires 2–3 days. However, collecting data from the NSO websites and implementing various data quality checks to finalize the data takes longer. Overall, the process takes around 1 month to complete.

For pillar 1 on data use, data is collected from four distinct sources. The World Bank supplies data for indicators on availability of comparable poverty data (from the World Bank’s Poverty and Inequality Platform (PIP) system (World Bank, 2022)^22^), and the indicator on quality of debt service data (from the World Bank’s *World Development Indicators* (WDI) metadata (World Bank, 2022b)^23^). Data on the availability of under-five child mortality data comes from the UN Inter-agency Group for Child Mortality Estimation. The indicator on availability of safely managed drinking water data is sourced from the WHO/UNICEF Joint Monitoring Programme. The indicator on the availability of source data for measuring labor force participation comes from the International Labour Organization (ILO). Each of these sources will be updated annually prior to each annual data release. The date the data is updated for each of these sources is available in our technical documentation.

For pillar 2 on data services, information on data dissemination subscription is collected from the International Monetary Fund (IMF)’s Dissemination Standards Bulletin Board. This and the WDI metadata follow the same update schedule and the release these two sources are identical. The online access indicator is sourced from the Open Data Watch’s Open Data Inventory (ODIN) openness score. The date of the data download is available in the technical description for this indicator. The indicator for data access services is based on (i) whether a portal is available, (ii) compliant with the Data Documentation Initiative (DDI) and with Dublin Core’s Resource Description Framework (RDF) metadata standards, and (iii) it has a listing of surveys and microdata sets that can provide the necessary data and reference for follow-up. This information is collected manually by visiting each NSO website.

For pillar 3 on data products, indicators are generated using the UN Global SDG monitoring database. For each SDG indicator, the database is checked to see whether a value is available within a five year window (for instance, for 2019 if a value is available between 2015–2019). For OECD countries, the UN SDG database is supplemented with comparable data submitted to the OECD following the methodology in “Measuring Distance to the SDG Targets 2019: An Assessment of Where OECD Countries Stand” (OECD, 2019)^24^. The decision to supplement the UN Global SDG monitoring database using this OECD database was taken, because a clear methodology had been established to do so. The UN Global SDG monitoring database was chosen as a primary source, rather than individual NSO websites, because data submitted to the UN Global SDG monitoring database goes through a standardized process including quality control and detailed documentation.

For pillar 4, on censuses and surveys, two complementary approaches are taken to collecting data. The first is to make use of data submitted to the World Bank, International Household Survey Network (IHSN), ILO, and FAO microdata libraries. Only surveys that are marked as nationally representative are used, and surveys are classified (as either health surveys, agriculture surveys, labor force surveys, etc.) using the classifications submitted to the microdata libraries. The contents of searches on these databases are available in the *github* repository. The second approach is a manual data collection effort, where NSO websites have been visited to be sure no surveys were missed. To be included in this search, the survey or census must be publicly available and accessible. This means at a minimum a survey report must be available detailing whether the survey was nationally representative and the start dates and completion dates of the field work. If surveys or censuses are missed in this search, the easiest way for a country to have it included would be to create an entry for the survey at one of the microdata libraries. Information on the completeness of the Civil Registry and Vital Statistics (CRVS) is sourced from the UN SDG global database. Information on whether data is available at the 1st administrative level, for the geospatial indicator, is sourced from ODW, as is the data openness score.

For pillar 5, data on the legislation indicator and finance indicators (compiled by PARIS21) are pulled from the UN SDG global monitoring database. Indicators in the standards and methods pillar are sourced primarily through the IMF. Information on the system of national accounts in use and national accounts base year are sourced through the World Bank’s WDI metadata. Data for the business process indicator is sourced through the United Nations Industrial Development Organization (UNIDO) and the United Nations Economic Commission for Europe (UNECE).

While efforts are made to provide as much data comparability across countries as possible, there are a number of dimensions where data harmonization is still in progress. These include (i) two dimensions under pillar 5 (Data infrastructure) where indicators are internationally comparable for most, but not all, countries, and (ii) eight dimensions under pillars 1, 2, 4, and 5 where no comparable data are available. These dimensions are respectively highlighted in yellow and red in Supplementary Figure A.2 in Appendix A, with more detailed discussion being provided in the technical documentation (Dang *et al*., 2021)^21^. The final dataset currently includes 12 dimensions. We return to discuss further ideas for improving the data in a later section (Issues for Further Considerations).

In general, missing data for a particular country-year observation is not imputed. There is one exception. One is that data from ODIN on data openness and geospatial data is available in 2015, 2016, 2017, 2018, and 2020, but not 2019. For 2019 data, the 2018 value is imputed by carrying forward the 2018 value for 2019.

**Fig. 2:**
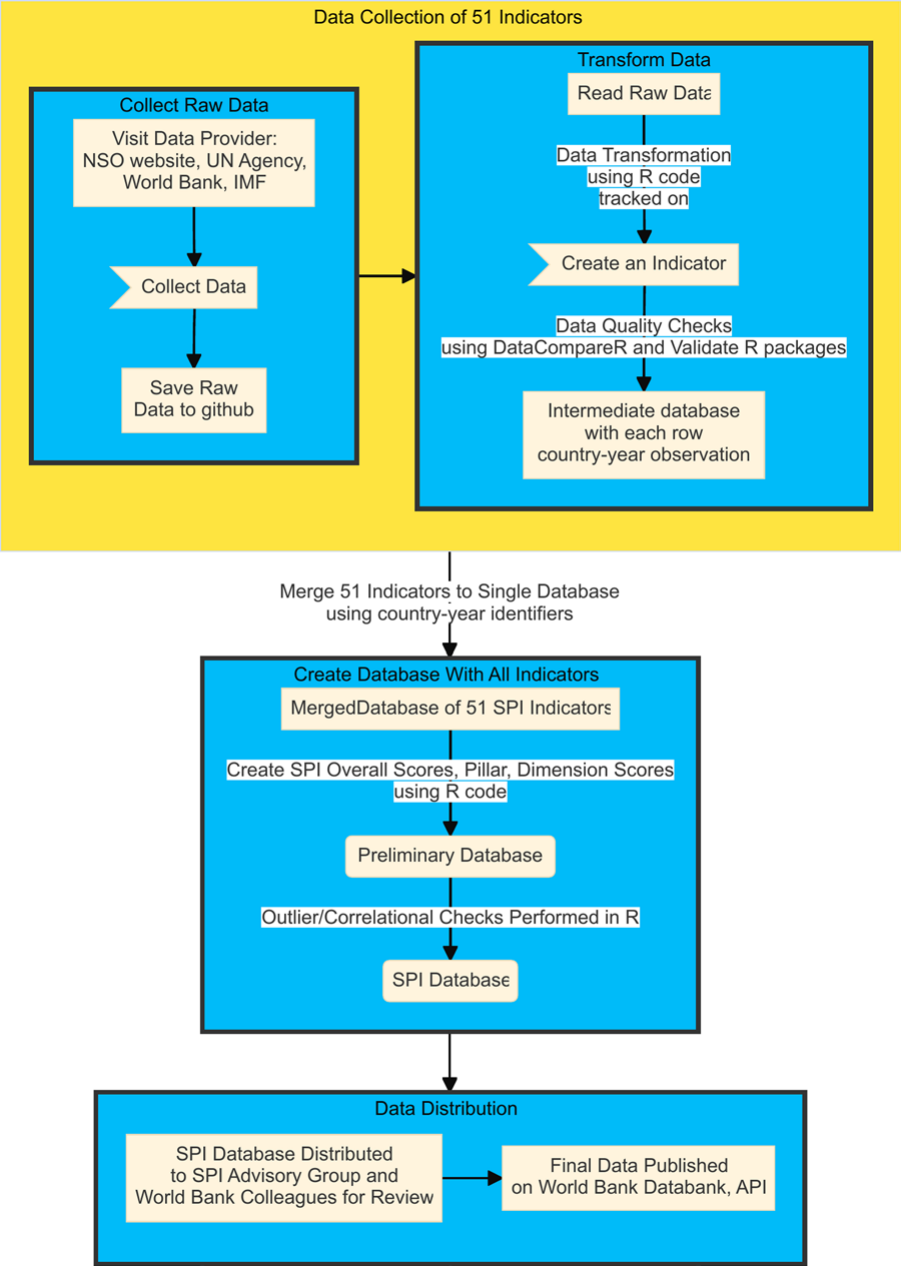
Data Flow Schematic. The final indicators making up the SPI are produced using the accompanying data flow. Raw data is collected from a variety of data providers. That data is then transformed, harmonized, and merged to a single file for distribution.

From these raw sources, the final indicators are created by transforming the raw information, so that each row in the dataset represents a unique observation for a country and year (e.g., Uganda in 2017, Mexico in 2019). Additionally, the 51 indicators are combined together into a single file by converting all country identifiers into a common format, which are the International Organization for Standardization (ISO)’s three alphabetic character country identifiers (*iso3c*). The process for transforming the raw information described above into a data base containing all 51 indicators for each country-year observation is described in Fig. 2.

### SPI Country scores

We provide full overall scores and pillar scores for all countries in 2020 in Supplementary Table A.2 (Appendix A).

**Fig. 3:**
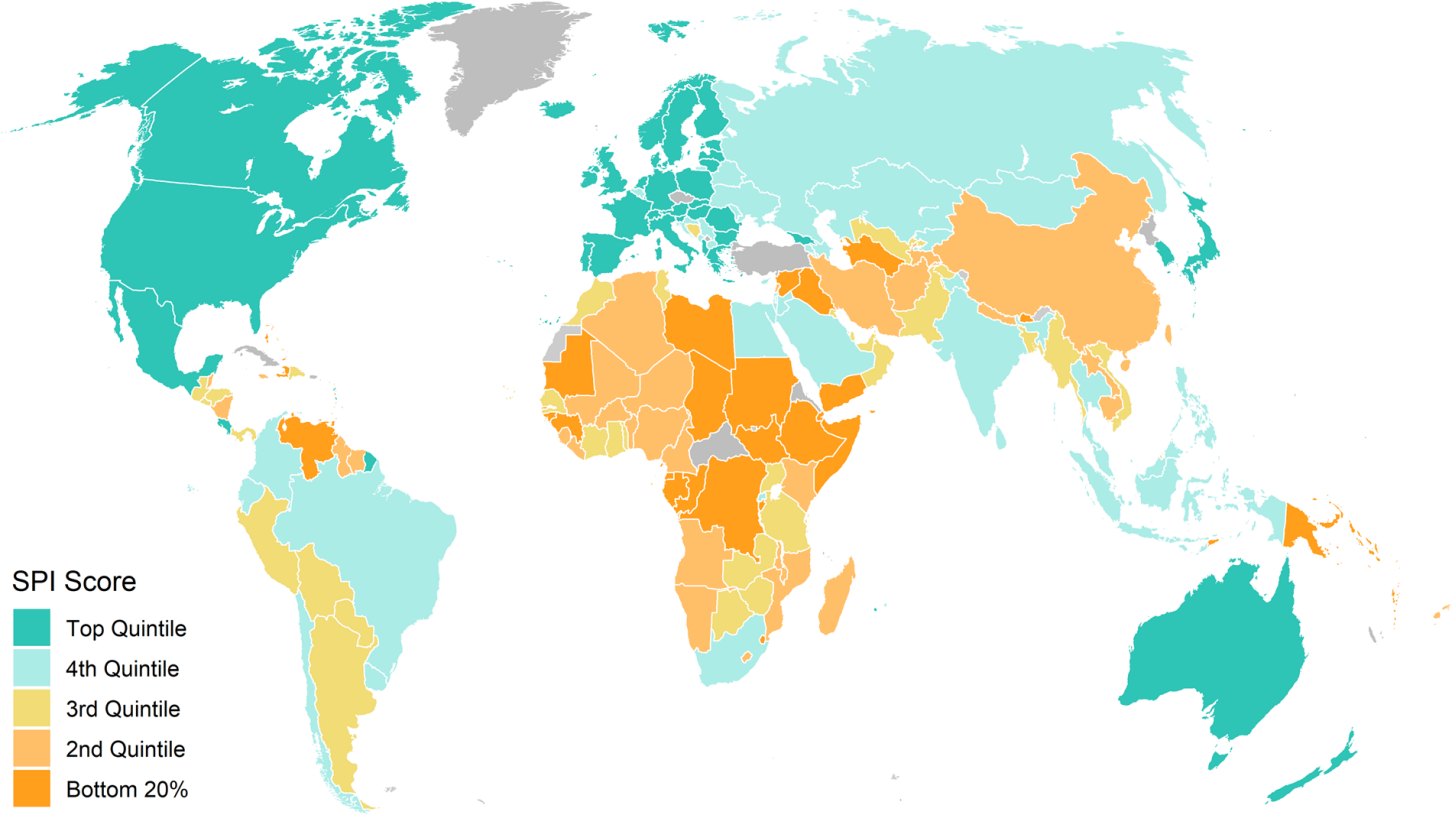
SPI Overall Scores. Shading for each point based on the quintile of the SPI overall score in 2020.

**Fig. 4:**
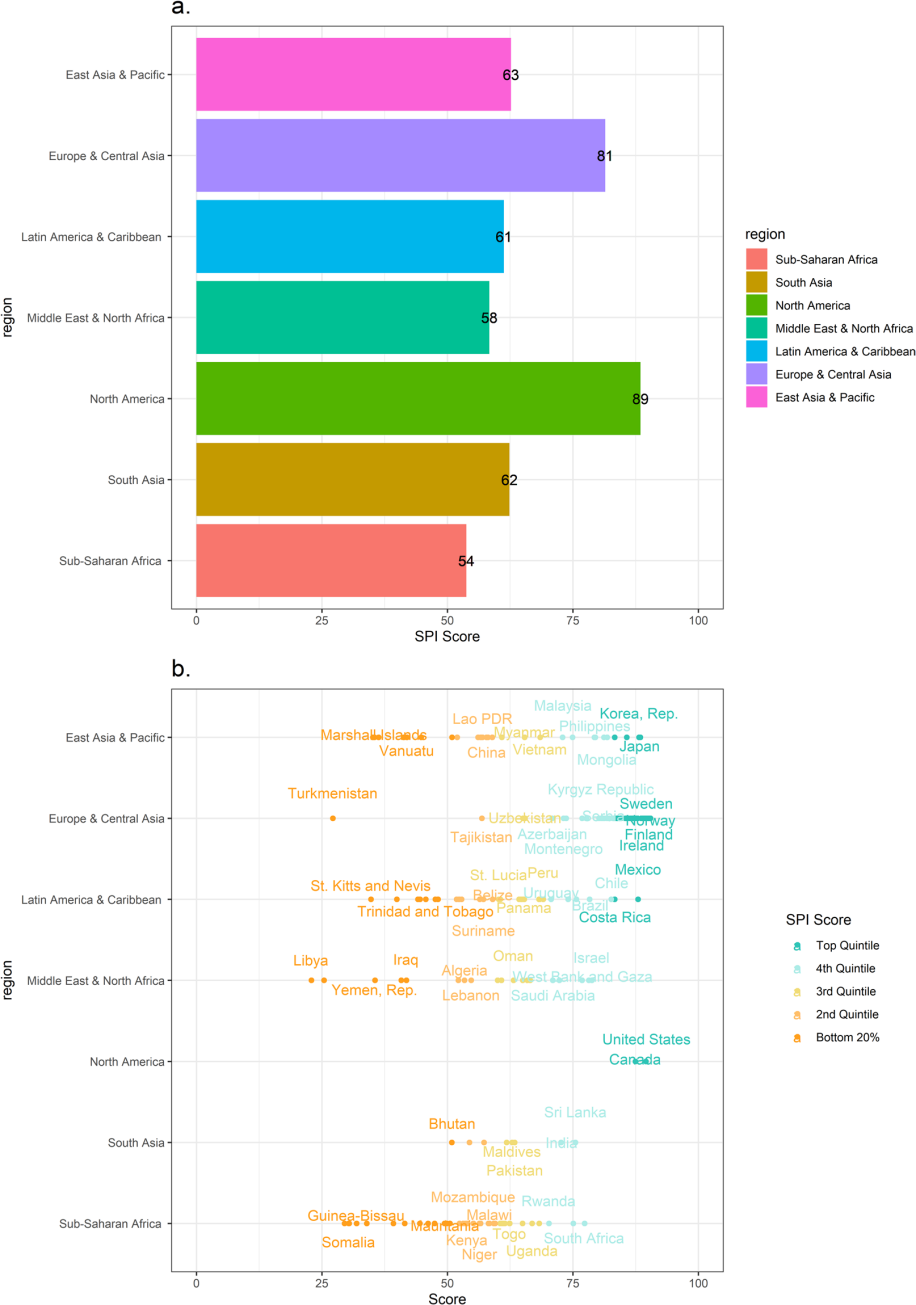
SPI Overall Score by Region and Country. (**a**) Regional averages of the SPI overall score in 2020. (**b**) Individual country SPI overall scores in 2020. Points shaded based on the quintile of the SPI overall score.

We map in Fig. 3 the SPI scores for all countries, which shows much heterogeneity for countries. Figure 4, Panel A further shows that there are large differences across regions. North America is the region with the strongest average SPI (89), which is followed by Europe and Central Asia (82), East Asia and the Pacific (63), South Asia (62), Latin America and the Caribbean (61), Middle East and North Africa (58), and Sub-Saharan Africa (54).

Yet, Fig. 4, Panel B shows that within regions there is also significant variation in the SPI overall score. For instance, in the Latin America & Caribbean region, Mexico is the country with the highest SPI score of 88, while Haiti, one of the lowest-scoring country in the region, earns a far lower score of 39.9. In Sub-Saharan Africa, the highest-scoring country is South Africa with a score of 77.3, while the lowest-scoring country is South Sudan with a score of 29.5. In the East Asia and Pacific region, the top-scoring country is Japan with a score of 88.4, while the lowest-scoring country is Kiribati with a score of 35.2.

We provide full overall scores and pillar scores for all countries in 2020 in Supplementary Table A.2 (Appendix A). Country SPI overall scores over the period 2016–2019 are provided in Dang *et al*.(2021)(Appendix B, Table B.2). To provide a visual aid with interpretations, the countries are color-coded into five groups based on their performance.

## Data Records

We uploaded a static version of the final SPI dataset (in Excel) on Figshare that includes the 51 indicators, sub-indicators, and the constructed indices (Dang *et al*., 2022^25^). This dataset, which is named “*SPI_data_code”*, is in a zipped folder and contains the final SPI dataset, along with all raw information collected to produce the indicators. The format of the raw information is varied, as it comes from several different sources including the IMF, NSO websites, the World Bank, ODW, several UN agencies, as well as other sources. Within the repository, the *01_raw_data* folder contains the raw data for the project for each indicator. This folder contains several subfolders linked to the 22 dimensions in our framework, with a readme file to help navigate the raw data folder. The raw information is available following a Creative Commons Attribution 4.0 license as well, which means users are free to share or adapt any of the materials available, so long as appropriate credit is given to the SPI team.

A live version of the data with annual updates, including the raw data, is available both on Github and publicly available in the World Bank’s DDH Data Catalog (Dang *et al*., 2022^25^). The index scores can also be downloaded using the World Bank API. The data is licensed under the Creative Commons Attribution 4.0.

Data is available for some indicators dating back to 2004, with index values available starting in 2016. In total, there are 181 countries with sufficient data to compute an index value. This set of countries covers 99.2 percent of the world population. We briefly describe the final *SPI_index* datafile below and provide a complete description of the variables in Supplementary Table A.3 (Appendix A).

*country*: country name

*iso3c*: country *ISO3* code

*date*: year of the data

*spi_index_pil1* to *spi_index_pil5*: SPI pillar scores 1 to 5

*spi_index*: SPI overall score

*spi_dim1_5_index* to *spi_d5_5_difi*: 52 indicator scores

*income*: any of 4 country income levels using World Bank classification, including low income, lower middle income, upper middle income, high income

*region*: any of 7 regions, including East Asia & Pacific, Europe & Central Asia, Latin America & Caribbean, Middle East & North Africa, North America, South Asia, and Sub-Saharan Africa

*population*: country population

## Technical Validation

### Data checks

The technical quality of the dataset is measured in a number of different ways. First, a set of automated data checks are performed, which involve comparing new values added to the dataset to the values of previous years to highlight unexpected changes or outliers and applying a set of data validation rules to check if any values of any columns are outside of expected bounds.

For new data added, the following validation checks are performed to highlight potential issues. First, the data set (*SPI_index.csv*) will be compared to the original dataset published on github. It is natural for some values to change from this vintage compared to last vintage, as the underlying data sources have been updated. However, this comparison helps to systematically monitor the differences and potentially highlight unexpected changes. The *dataCompareR* package is used for this purpose in *R* (Johnston *et al*., 2021^26^).

Second, the data set is compared to a set of expected values for each column. The exact expected values is listed in the appendix (Supplementary Information), but the general idea is to check that the values adhere to rational min/max values and positive correlate in expected ways. For instance, the availability of SDG indicators columns are the fraction of SDG indicators in a goal with a value in the previous 5 years. Thus, they should be between 0 and 1. Additionally, the countries and dates included are matched to a pre-specified expected list to uncover the accidental inclusion/exclusion of dates or countries. The *validate R* package is used for this purpose (van der Loo & de Jonge, 2021^27^). A code snippet containing the validation rules is available in the Supplementary Information, Appendix C.

Third, we examine the index using different weighting methods, which does not change the results significantly. For example, using a weight of 1/6 for censuses and surveys (instead of ¼ in pillar 4 on data sources) provides very similar results. In particular, the correlation between the SPI overall score under the preferred approach and the alternative approach is 0.998.

Finally, we provide qualitative data checks by regularly consulting with the SPI Advisory Group and colleagues who are experts at the World Bank and other international organizations (e.g., FAO, IMF, ILO) on different development subjects. In particular, we obtain data help through our long-running collaboration with colleagues at other international agencies (e.g., by asking them to reach out to their contacts in the NSOs for data verification or clarification). Similarly, wherever data for some indicators (countries) are in doubt, we also discuss with our country-based colleagues who are charged with daily monitoring of country development. This internal validation process is based on established data quality procedures that we implement for all the other databases produced by the World Bank such as the *World Development Indicators*.

### Comparison with SCI

Further comparison with the SCI is provided in Appendix C in the Supplementary Information, which confirms that the SPI covers more countries and more indicators. Most importantly, it distinguishes all the countries’ statistical performance with a unique score. It is also less volatile over time than the SCI.

### Further decompositions by pillars and income levels

**Fig. 5:**
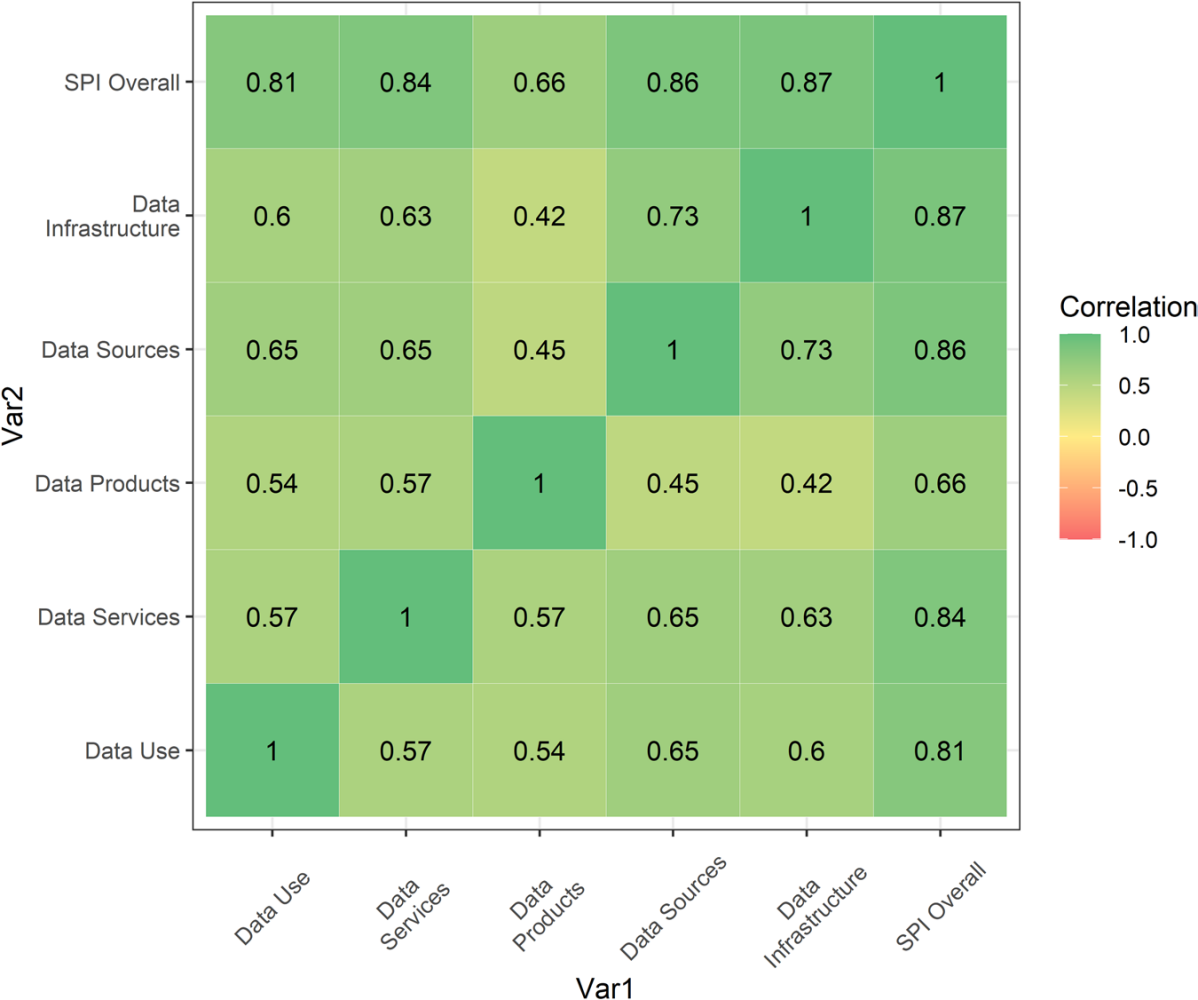
Correlation between the SPI Pillars. Pearson correlation coefficients for SPI pillar and overall scores in 2020.

We show next in Fig. 5 the correlations between the SPI overall score and the SPI pillar sub-scores (Data Use, Data Services, Data Products, Data Sources, and Data Infrastructure). All pillars are positively correlated with one another. On the other hand, no pillar is perfectly correlated with any of the other pillars, which indicate that each pillar provides additional information on a country’s statistical performance. The pillars with the correlation to the overall measure in a decreasing order is pillar 5 on data infrastructure (0.87), pillar 4 on data sources (0.86), pillar 2 on data services (0.84), and pillar 1 on data use (0.81). The indicator with the lowest overall correlation with the SPI overall score is pillar 3 on data products (0.66). We provide more visual illustration for the five data pillar scores globally and for countries within each region in Dang *et al*. (2021^14^, Appendix A).

**Fig. 6:**
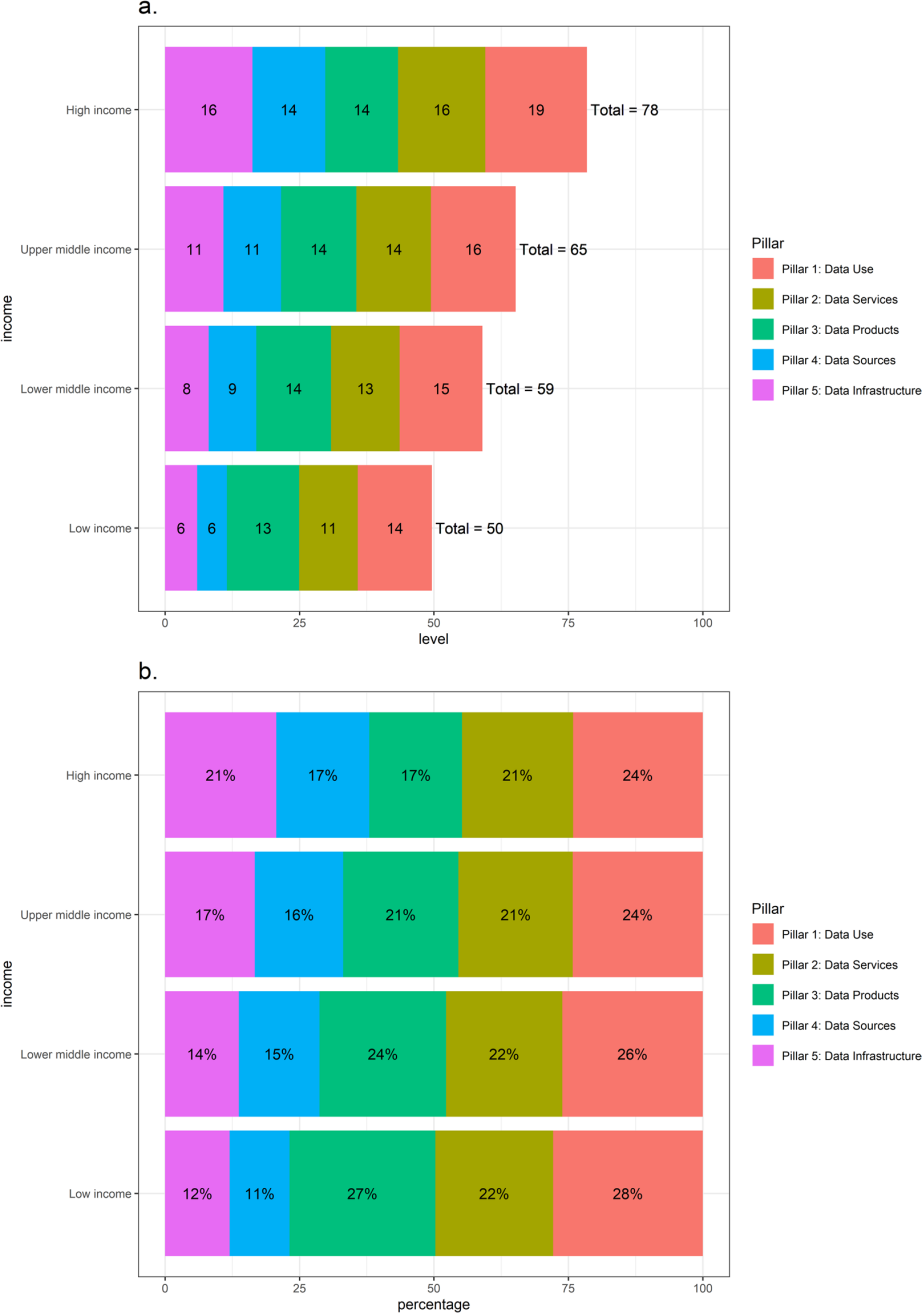
Contribution of Each Pillar to SPI Overall Score, by Country Income Level. (**a**) Contribution in absolute terms. (**b**) Contribution in relative terms. Data for the year 2020.

As discussed earlier, we can further decompose the SPI into the contributions from each pillar, in both absolute and relative terms, by country income level. In Fig. 6, Panel A, we show the decomposition in absolute terms for countries in each income level. For a hypothetical country that scores perfectly in each pillar, the figure would show 20 points for each pillar. In low income countries, adequate data sources (pillar 4) represents a severe capacity limitation: it contributes only 5.5 points out of the maximum 20 points for low income countries to the SPI overall score. Data infrastructure is another area of concern for low income countries, with low income countries receiving only 5.9 points, compared to 16.2 points for high income countries.

In relative terms, low income countries are doing comparatively well in terms of the data use pillar, with 27.7 percent of low income countries scores coming from this pillar, and relatively poor on data sources with only 11.9 percent of the overall score coming from this pillar. High income countries are doing relatively poorly in terms of data products with 17.2 percent of the overall score coming from pillar 3.

**Fig. 7:**
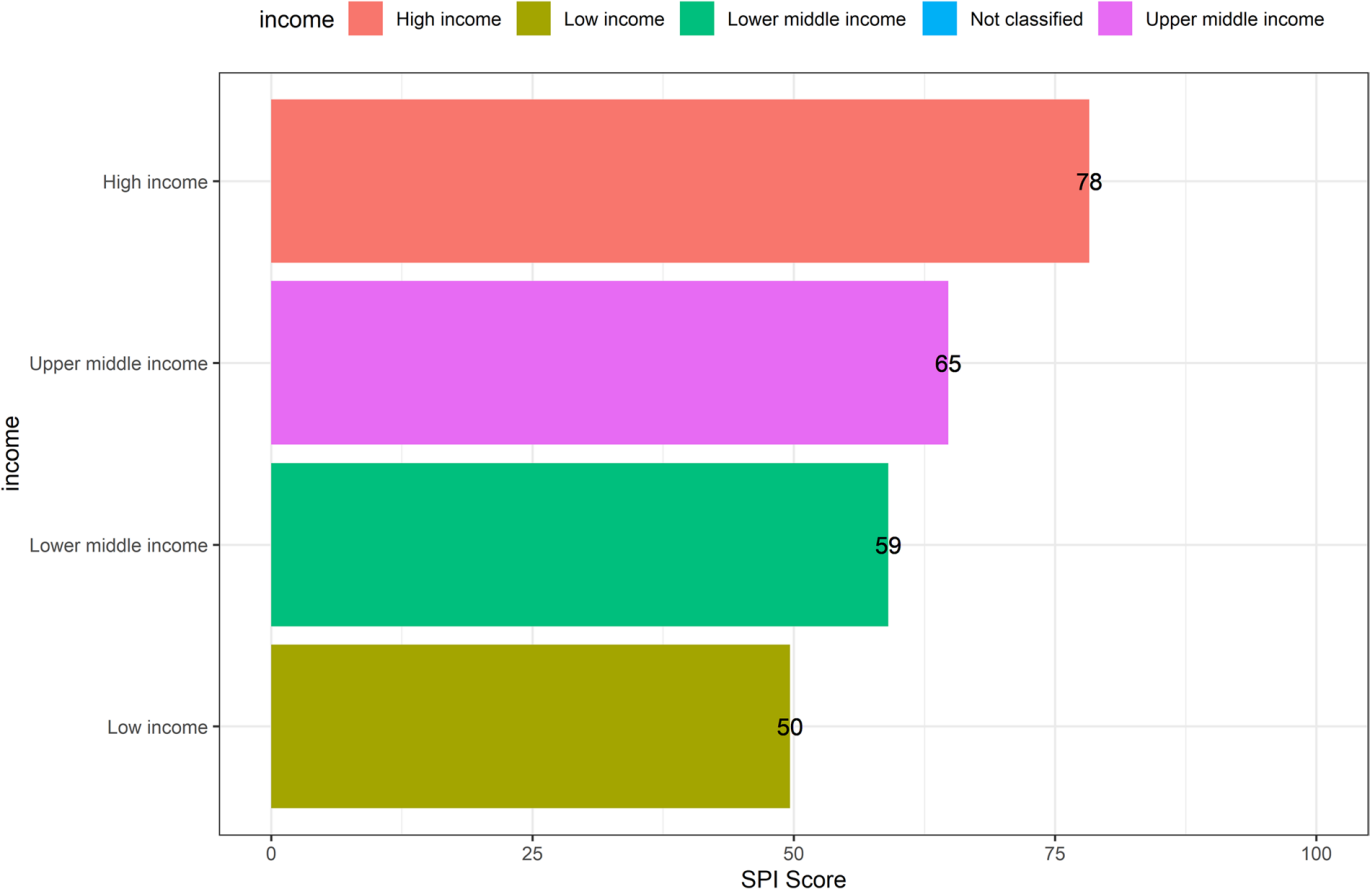
SPI Overall Scores by Income Group. Income group averages shown for the year 2020.

We examine the SPI in more detail by income levels in Fig. 7. This figure shows that countries with a higher income level have a higher SPI score. In particular, high income countries have an average SPI of 78, which is followed by upper middle income countries (66), lower middle income countries (59), and low income countries (49). In terms of relative differences, the SPI score for high income countries is 18 percent higher than that of upper middle income, 32 percent higher than that of lower middle income countries, and 59 percent higher than that of low income countries. Overall, the correlation in 2020 between (logged) GDP per capita and the SPI overall score is 0.6.

Using a related ranking of the SPI by World Bank’s country lending status shows a similar positive correlation between country income level and its SPI score. In particular, the SPI scores are lowest for IDA (poorest) countries and highest for unclassified (high-income) countries. Similarly, dividing countries into FCS (Fragile and Conflict) status versus non-FCS status respectively yields the scores of 69 and 46 for the former and latter groups of countries (results are available upon request).

## Usage Notes

### SPI in recent studies

Despite its infancy, to date the SPI has been adopted for measuring country statistical capacity in several global policy reports such as *Sustainable Development Report 2021* (Sachs *et al*., 2021)^28^ and World Bank’s *World Development Report 2021* (World Bank, 2021)^29^. It was used to help highlight various gaps in countries’ data dissemination and openness, with higher statistical capacity levels being positively correlated with more NSO independence (World Bank, 2021)^29^. The index has motivated further research on assessing data openness and accessibility in MENA, a traditionally data-scarce region (Ekhator-Mobayode and Hoogeveen, 2021)^30^, or how best to construct measures for learning deficiency due to Covid-19-induced school closures (Azevedo, 2020)^31^. It also contributes to current thinking on topics ranging from improving the quality of NSSs and future official statistics (Radermacher, 2021^32^; Bersales, 2022^33^) and food and agriculture statistics (Bizier *et al, 2022*.^34^) to selecting the appropriate context to measure student absenteeism and women’s empowerment (Evans and Acosta, 2021^35^; Yount *et al*., 2022^36^).

### Issues for further consideration

We briefly discuss several existing limitations as well as promising directions to expand the SPI and its practical relevance for policy recommendations.

First, more understanding is needed of how statistical data are used. Some promising work has been done by PARIS21 on data use by the Executive Branch of Government and on data use by citizens but there is an important research agenda to be pursued. This research can lead to fruitful policy advice. For example, there is evidence that using volunteer data collected by citizens can encourage the public to participate more in environmental protection and enhance government ability to monitor and manage natural resources and public service delivery (Conrad and Hilchey, 2011^37^; Meijer and Potjer, 2018^38^). A study on 10 Latin American countries further suggests that more demand for high-quality statistics could provide an important incentive for a country’s statistical capacity to improve (Dargent *et al, 2018*.^39^). Statistics have no value unless they are used and it is only through an understanding of how they are used, the extent of that use and drivers for better use that statistical systems can be designed in a user-centered way. In this regard, we acknowledge that conceptually, while the new SPI is intended to provide the world with a new forward looking framework of how NSSs need to further evolve, the SPI scores are empirically based on the data currently available. As such, it must be further refined, based on collective investment in developing more relevant measurements and data sources.

Second, the United Nations Statistics Division global database for SDG indicators is not well populated, particularly for many high-income countries. (Notably, high-income countries do not often collect data on certain indicators that are more relevant for poorer countries such as child stunting, so they do not report on these indicators to the SDGs). A recent study suggests that data are available for just over half of all indicators and for just 19 percent of what is needed to comprehensively track progress across countries and over time (Dang and Serajuddin, 2020)^40^. Data gaps for vulnerable groups including women and girls are even more challenging, with only 10 of the 54 gender-specific SDG indicators being widely available and up to international standards (UN Women, 2018)^41^. Data gaps for women are further worsened during the Covid-19 pandemic, which more negatively affected women. For example, fewer than half (41 percent) of poorer countries report data on informal jobs—where women predominate—that are disaggregated by gender and an even smaller proportion (38 percent) of SDG economic opportunity indicators are available by gender in the most pandemic-affected countries (Buvinic *et al*., 2020)^42^.

In some cases it is likely that the data exists but has not found its way on to the database. This is a major problem for users and for those seeking to identify best country practice as a guide for their own statistical development. This issue is related to serious gaps in information on data sources and also applies across the range of administrative data sources. It would be important for the United Nations custodian bodies to work with countries to improve this situation. Statistics about statistics are vital if improvements to statistical systems are to be made.

Third, data about statistical infrastructure is patchy. In particular it will be useful to collect further data on the financing of statistical systems. One of the Sustainable Development Goal indicators (17.18.3) relates to whether the national statistics plan is fully funded but there remains incomplete data on this. Financing is also a complex issue since the costs that are associated with producing high-quality and more frequent data vary across countries and are likely to reflect, at least to some extent, country statistical capacity. For instance, a study suggests that the average cost of implementing a recent household consumption survey (in 2014 or later) ranges from approximately US$800,000 to US$5 million, depending on the context and sample sizes (Kilic *et al*., 2017)^43^. Countries with a better data infrastructure (and higher levels of data skills) may possibly offer lower costs regarding data collection. Furthermore, research into the different strengths of correlation between the five data pillar scores can be useful. For example, does a stronger correlation of the overall SPI score with data infrastructure justify prioritized investment in this area?

Related to this, certain countries heavily rely on donor funds and technical assistance to implement data collection activities (e.g., household surveys). Everything else equal, these countries likely have lower statistical capacity than countries that implement surveys on their own. As such, it is useful to investigate further how each country produces its data and whether some aid-dependent countries, in a counterfactual scenario, can continue their statistical activities without interruptions in the absence of donors. Such scenarios are not far-fetched. Donors such as the Swedish Aid Agency (SIDA) or the United Kingdom Foreign, Commonwealth and Development Office (FCDO) are known in the past decade to have withdrawn resources from developing countries that have become richer (e.g., Vietnam) to focus more on the poorer countries (e.g., Sub-Saharan Africa). An accurate evaluation of a (poor) country’s NSS statistical capacity is thus quite helpful for the donor and the government to discuss alternative aid strategies when this country raises its income level.

In the long term, it is both useful and beneficial for NSOs to further participate in providing data on data infrastructure (as well as data use). Put differently, stronger engagement from NSOs in various stages of the process—from data collection to data analysis and dissemination—can serve not only as the means to improve data quality, but also as the end objective of improving country statistical capacity. Such collaboration between local NSOs and the World Bank can be started on an experimental basis as with most development projects, with a view toward subsequent scaling up on a regional basis and finally on a global scale.

More generally, ongoing technological advances with computing and data storage power have made various data sources more accessible. The cycle of data collection activities has also significantly been shortened, due to decreasing costs of mobile devices and faster internet connections. In this regard, the roles of NSS may increasingly transition from the more basic tasks of data collection and dissemination to the more advanced tasks of data analysis and interpretation. Alternative methods of producing data, such as employing statistical data imputation methods, have been found to substitute for the need to collect data at least to some extent (Dang, Jolliffe, and Carletto, 2019)^44^. Consequently, future updates of the SPI can include more indicators for an NSO’s analytical capacity to produce various statistical products.

Finally, a question can be raised on how often the SPI’s framework (including its indicators) should be updated? For example, should it be on a five-year basis or a shorter basis? Past experience with the SCI suggests that this index has served multiple objectives well in the past two decades. But this took place in a less changing data landscape, which used to have less technology development and more expensive data equipment. Just in the past few years, we have seen all the constraints loosened with fast technological progress as discussed earlier. While we do not offer conclusive thoughts on this topic, it may be useful to open up the discussion with various stakeholders on the best ways to ensure that the SPI remains useful and relevant in a fast-changing world.

## Supplementary information

Supplementary Information

## Data Availability

The SPI Github repository contains the raw data used to produce our indicators, code to reproduce the values for all of our indicators and overall scores written in *R*, which is an open source statistical language, and a final data set available in CSV and Stata format. A detailed Readme file is available detailing how to use the code. The repository is licensed under the Creative Commons Attribution 4.0 International License, which means users are free to share or adapt any of the materials available, so long as appropriate credit is given to the SPI team. The Github repository also contains the version control history, which documents every change in the data and code of the entire project dating back to July 2020 to build confidence and transparency. The vast majority of code in this repository is written in the *R* language. The *R* version used was 4.0.3 (2020-10-10). This repository contains several files from the *R* package “*renv*”^45^. The *renv* package helps manage specific package versions used to produce the results in this repository. Because package version conflicts can make code that runs on one system not run on another system, it is important to have a list of the specific package versions used and a workflow for accessing these specific packages. The *renv* package provides this. In general, the *renv::restore()* command should install all packages found in the *renv.lock* file in this repository, so that version conflicts do not cause errors. An SPI Interactive Dashboard is available for a more detailed exploration of the data. In this application, it is possible to map any of the 51 Statistical Performance Indicators, to explore a country report for each of the 174 countries, and use a tool to see how the SPI overall scores change when alternative weights are applied to the dimensions and pillars. **Version update** This paper builds on but significantly expands the earlier analysis in the (unpublished) working documents by Dang *et al*. (2021)^14^ and in Dang *et al*. (2021)^21^. All the sections are new, except for the three subsections “Conceptual Motivations”, “Construction of the SPI”, and “Issues for Further Considerations”, which are revised and updated with more recent references. This paper analyzes new SPI data for 2020, while the two cited references analyze data up to 2019. The appendix materials in the Supplementary Information are mostly based on these two cited references, except for the updated discussion related to the 2020 SPI data.
