# Supplementary material for: Statistical performance indicators and index—a new tool to measure country statistical capacity
Source: Sci Data. 2023 Mar 20;10:146. doi: 10.1038/s41597-023-01971-0 (PMC10025805; doi:10.1038/s41597-023-01971-0)
Supplement: Supplementary file 1 — Supplementary Information [file 41597_2023_1971_MOESM1_ESM.docx]

# Supplementary Information

***********

**Statistical Performance Indicators and Index—A New Tool**

**to Measure Country Statistical Capacity**

Hai-Anh H. Dang

World Bank; International School, Vietnam National University, Hanoi; Indiana University; IZA; GLO

([hdang@worldbank.org](mailto:hdang@worldbank.org))

John Pullinger

United Kingdom National Statistical Service

Umar Serajuddin

World Bank

Brian Stacy

World Bank

This Supplementary Information includes the following Appendixes

1. Appendix A: SPI Description and Scores
   1. Part A. SPI Pillars and Dimension
   2. Part B. SPI Scores in 2020
2. Appendix B: Useful Properties of the SPI
3. Appendix C: Further Comparison with the SCI

# Appendix A: SPI Description and Scores

## Part A. SPI Pillars and Dimension

A quick primer on names. We refer to the 5 rows in the framework in Figure 1 as pillars. We refer to the 22 cells in the framework in Figure 1 as dimensions. Finally, each dimension may be composed of multiple indicators. For instance, the dimension on censuses and surveys is made up of indicators on whether population censuses have been conducted, agriculture censuses, labor force surveys, etc.

**3.1 Data use**

The data use pillar is segmented by user type. The tiles on the Dashboard provide an indicator of use of statistics respectively by the legislature, executive, civil society (including sub-national actors), academia and international bodies. A mature system would score well across the tiles. Areas for development would be highlighted by weaker scores in that domain enabling questions to be asked about prioritization among user groups and why existing services are not resulting in higher use of national statistics in that segment.

**3.2 Data services**

The data services pillar is segmented by service type. The tiles on the Dashboard provide an indicator of the quality of data releases, the richness and openness of online access, the effectiveness of advisory and analytical services related to statistics and the availability and use of data access services such as secure microdata access. Advisory and analytical services might incorporate elements related to data stewardship services including ethical consideration of proposals and calling out misuse of data in accordance with the Fundamental Principles of Official Statistics.

**3.3 Data products**

The data products pillar is segmented by topic and organized into social, economic, environmental and institutional domains using the typology of the Sustainable Development Goals. This approach enables comparisons across countries and anchors the system in the 2030 agenda so that a global view can be generated while enabling different emphasis to be applied in different countries to reflect the user needs of that country.

**3.4 Data sources**

The data sources pillar is segmented between sources generated by the statistical office (censuses and surveys) and sources accessed from elsewhere (administrative data, geospatial data, private sector data and citizen generated data). The appropriate balance between these types of sources will vary depending on the institutional setting and maturity of the statistical system in each country. High scores should reflect the extent to which the sources being utilized enable the necessary statistical indicators to be generated. For example, a low score on environment statistics may reflect a lack of use of (and low score for) geospatial data. This linkage, which is inherent in the data cycle approach, should help highlight areas for investment if country needs are to be met.

**3.5 Data infrastructure**

The data infrastructure pillar is segmented into hard and soft infrastructure segments itemizing essential cross-cutting requirements for an effective statistical system. The segments are:

1. Legislation and governance covering the existence of laws and a functioning institutional framework for the statistical system
2. Standards and methods addressing compliance with recognized frameworks and concepts
3. Skills including level of skills within the statistical system and among users (statistical literacy)
4. Partnerships reflecting the need for the statistical system to be inclusive and coherent
5. Finance, both domestically and from donors

### Supplementary Table A.1: Description of SPI Dimensions

| Dimension | Brief Description |
| --- | --- |
| Dimension 1.1: Data use by national legislature | Not included because of lack of established methodology. In principle it may be possible to utilize websites of national legislatures but this will require further work and assessment. |
| Dimension 1.2: Data use by national executive branch | Not included because of lack of established methodology. There are some usable data sources (as used by (PARIS21 2019)) but gaps in data across countries have prevented full adoption. |
| Dimension 1.3: Data use by civil society | Not included because of lack of established methodology. There are some usable data sources with good coverage, for example from social media but more data is required to help assess and allow for likely biases between and within countries. |
| Dimension 1.4: Data use by academia | Not included because of lack of established methodology. We have not been able to find usable data sources with global coverage on which a new methodology could be developed. |
| Dimension 1.5: Data use by international organizations | Five measures usefulness or reliability of country produced measures for international organizations have been included. First, on comparability of poverty estimates for the World Bank reporting on international poverty using [Povcalnet](http://iresearch.worldbank.org/PovcalNet). Second on usable surveys for statistics on child mortality for the [UN Inter-agency Group for Child Mortality Estimation](https://childmortality.org/). Third on accuracy of debt reporting as classified by the World Bank (Source: World Bank WDI metadata). Fourth, on availability of safely managed drinking water data for use by [WHO/UNICEF Joint Monitoring Programme](https://washdata.org/). Fifth, on labor force participation data for use by ILO. While these data sources provide only a partial coverage of data used by international organizations, they do provide an indication of the performance of the national statistical system. |
| Dimension 2.1: Data releases | SDDS/e-GDDS subscription. This indicator is based on whether the country subscribes to IMF SDDS+, SDDS, or e-GDDS standards. The source is the IMF Dissemination Standards Bulletin Board. This is a reliable data source but we recognize that it is a proxy for the concept we are seeking to capture rather than a direct measurement. |
| Dimension 2.2: Online access | ODIN Open Data Openness score (Jamison Crowell et al. n.d.). This is a well-established data source with good country coverage, which scores countries based on whether indicators are available online in a format that is machine readable, in a non-proprietary format, downloadable, with metadata available and terms of use. Scores range from 0-1. For more details, consult the [ODIN technical documentation](https://docs.google.com/document/d/1MBK0hN6MoQrii7_E1bmRXmsUcE8Fbb-Q32nxm8d8qTw/edit) |
| Dimension 2.3: Advisory/ Analytical Services | Not included because of lack of established methodology. This could be a new indicator of the number of non-recurring products on NSO website (ad hoc/experimental rather than regular releases). The indicator is the number of products found. No established source exists for this indicator. |
| Dimension 2.4: Data access services | NADA metadata. This indicator checks whether NADA microdata cataloging is available for surveys produced by NSO. NADA is an open source microdata cataloging system, compliant with the Data Documentation Initiative (DDI) and Dublin Cores RDF metadata standards. Source: NSO websites. |
| Dimension 3.1: social statistics | Availability of Goal 1-6 indicators, measured by an average score. The primary data source is the UN SDG database. While this is a database with comprehensive coverage that all countries have signed up to, many countries are not yet submitting all their available national data. Scores for some countries thus may not capture their performance in calculating the indicators. For OECD countries, we supplement the UN SDG database with comparable data submitted to the OECD following the methodology in [Measuring Distance to the SDG Targets 2019: An Assessment of Where OECD Countries Stand](https://www.oecd.org/sdd/measuring-distance-to-the-sdg-targets-2019-a8caf3fa-en.htm). |
| Dimension 3.2: economic statistics | Availability of Goal 7-12 indicators, measured by an average score. See 3.1. |
| Dimension 3.3: environmental statistics | Availability of Goal 13 & 15 indicators, measured by an average score. Goal 14 - Life on Water - is not included because land-locked countries do not report on these indicators. See 3.1. |
| Dimension 3.4: institutional statistics | Availability of Goal 16-17 indicators measured by an average score. See 3.1. |
| Dimension 4.1: censuses and surveys | Availability of recent censuses and surveys covering broad areas. The following censuses and surveys are considered: Population & Housing census, Agriculture census, Business/establishment census, Household Survey on income/ consumption/ expenditure/ budget/ Integrated Survey, Agriculture survey, Labor Force Survey, Health/Demographic survey, Business/establishment survey. Source: NSO websites, World Bank microdata library, ILO microdata library, IHSN microdata library |
| Dimension 4.2: administrative data | Availability of Civil Registration and Vital Statistics (CRVS) indicator. An ideal indicator would include a score based on the density of administrative data available in sectors of social protection, education, labor, and health. However, social protection, education, health, and labor admin data indicators not included because of lack of established methodology. While several promising sources for administrative data from the World Bank’s ASPIRE team, WHO, UNESCO, and ILO have been identified, they were not included due to incomplete coverage across countries. Further research and data collection effort would be needed to fill in this information, so that a more comprehensive picture of administrative data availability can be produced. |
| Dimension 4.3: geospatial data | Geospatial data available at 1st Admin Level. This data source from Open Data Watch focusing on data availability at the sub-national level provides a partial understanding of a country’s ability to produce geospatial data. A research and data collection effort is needed to develop a more comprehensive global database of the availability of key geospatial indicators. |
| Dimension 4.4: Private/citizen generated data | Not included because of lack of established methodology. Currently no comprehensive source exists to measure the use of private and citizen generated data in national statistical systems, and this should be another area where more data collection is needed by the international community. |
| Dimension 5.1: Legislation and governance | This indicator is based on PARIS21 indicators on SDG 17.18.2 (national statistical legislation compliance with UN Fundamental Principles of Official Statistics), existence of National Statistical Council, national statistical strategy generation, national statistical plan. Limited country coverage makes cross country comparison limited. So this is included in the dashboard, but not in the overall SPI score or index. |
| Dimension 5.2: Standards and Methods | This set of indicators is based on countries’ use of internationally accepted and recommended methodologies, classifications and standards regarding data integration. These indicators help facilitate data exchange and provide the foundation for the preparation of relevant statistical indicators. The following methods and standards are considered: System of national accounts in use, National Accounts base year, Classification of national industry, CPI base year, Classification of household consumption, Classification of status of employment, Central government accounting status, Compilation of government finance statistics, Compilation of monetary and financial statistics, Business process. Further work could improve the validity of this indicator and reduce the risk that countries may be incentivized to adopt only traditional standards and methods and neglect innovative solutions that may be more valid in the current context. |
| Dimension 5.3: Skills | Not included because of lack of established methodology or suitable data sources. A new indicator drawing on PARIS21 indicators such as statistical society presence and data literacy could be developed and is an area of future work. |
| Dimension 5.4: Partnerships | Not included because of lack of established methodology or suitable data sources. A new indicator based on textual analysis of NSS reports/websites for references to partner organizations could be developed. This is an area of future work. |
| Dimension 5.5: Finance | The indicator is based on PARIS21 SDG indicators (SDG 17.18.3 (national statistical plan that is fully funded and under implementation). It is included in dashboard, but not in the overall SPI score or index because of insufficient country coverage. |

### Supplementary Figure A.1: Virtuous Data Cycle


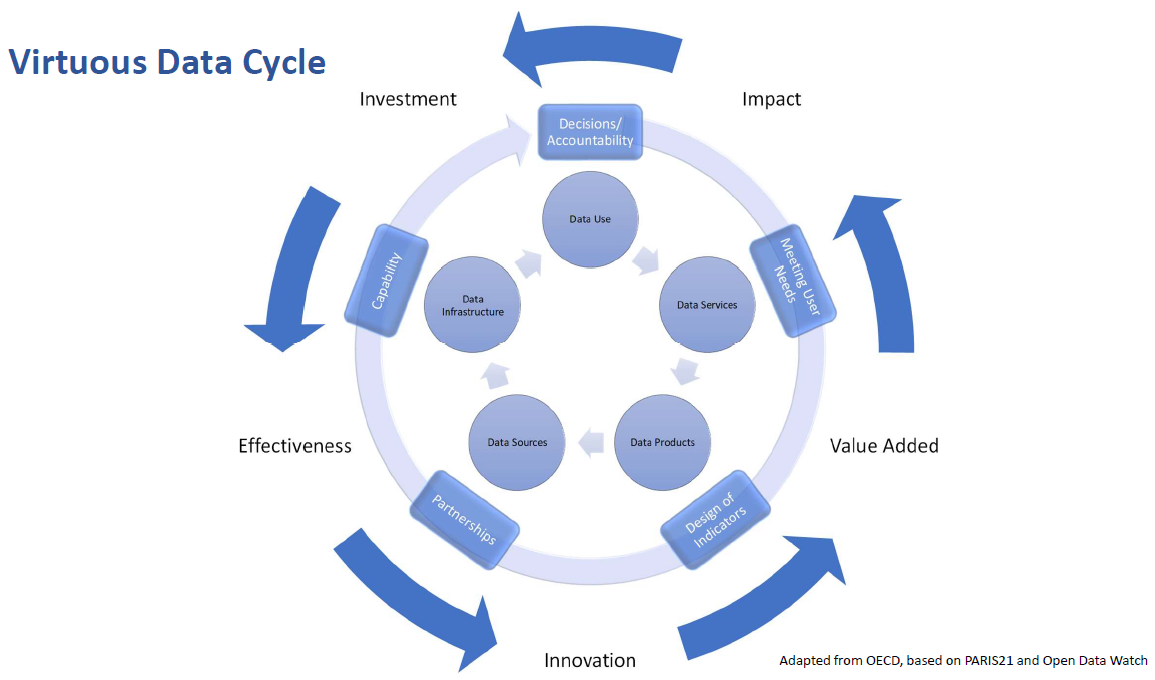


### Supplementary Figure A.2: Missing Elements in the SPI Framework


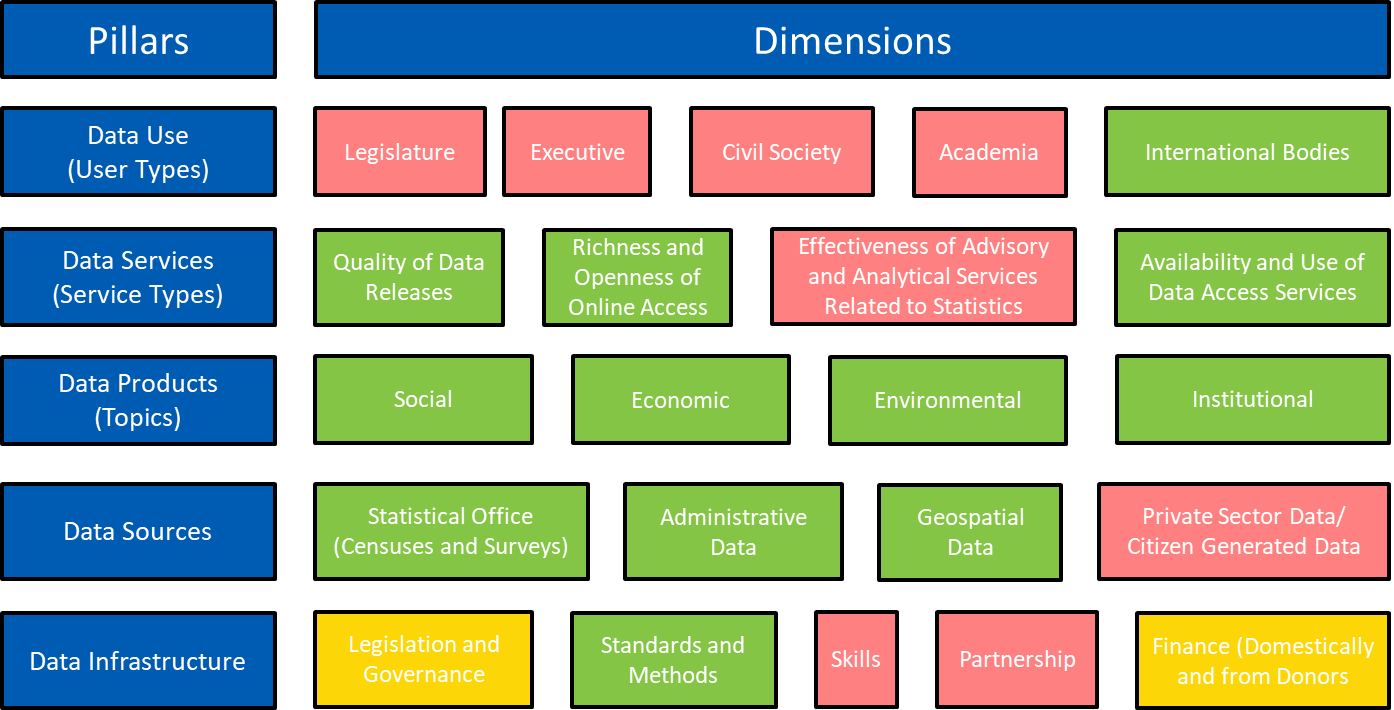


*Notes: Green tiles show areas with internationally comparable indicators for nearly all countries. Yellow tiles show areas with internationally comparable indicators for most, but not all, countries. Red tiles show areas with no comparable data available. More detailed discussion is provided in* (Dang, et al., 2021)**^Error! Reference source not found.^**.

## Part B. SPI Scores in 2020

Below, the full list of countries by their SPI overall score in 2020 is presented. The first column is the country name and the following columns are the overall SPI overall score, and then the sub-scores for pillars 1, 2, 3, 4, and 5.

The purpose of the SPI is to help countries assess and improve the performance of their statistical systems. The presentation of SPI overall scores is designed to reflect that aim. Small differences between countries should not be stressed since they can reflect imprecision arising from the currently available indicators rather than meaningful differences in performance. Instead, the presentation of overall SPI scores focuses on larger groupings of countries reflecting broad categories of performance as measured by the indicator framework. In total there are 174 countries with sufficient data to compute an index value. This set of countries covers 99.2 percent of the world population.

Countries shaded in dark orange are the lowest performing, countries in dark green are the highest performing. Countries are grouped into five groups:

1. **Top Quintile**: Countries in the top 20% are classified in this group. Shading in dark green.
2. **4th Quintile**: Countries in the 4th quantile, or those above the 60th percentile but below the 80th percentile are in this group. Shading in light green.
3. **3rd Quintile**: Countries in the 3rd quantile, or those between the 40th and 60th percentile, are classified in this group. Shading in yellow.
4. **2nd Quintile**: Countries in the 2nd quantile, or those above the 20th percentile but below the 40th percentile, are in this group. Shading in light orange.
5. **Bottom 20%**: Countries in the bottom 20% are classified in this group. Shading in dark orange.

### Supplementary Table A.2: SPI overall score and Pillar Scores in 2020

| Country | SPI overall score | Pillar 1: Data Use | Pillar 2: Data Services | Pillar 3: Data Products | Pillar 4: Data Sources | Pillar 5: Data Infrastructure |
| --- | --- | --- | --- | --- | --- | --- |
| Norway | 90.5 | 100.0 | 94.3 | 79.1 | 78.9 | 100 |
| Finland | 90.1 | 100.0 | 95.7 | 79.2 | 75.7 | 100 |
| Austria | 89.8 | 100.0 | 92.0 | 79.4 | 77.4 | 100 |
| Italy | 89.6 | 100.0 | 92.2 | 77.7 | 78.2 | 100 |
| Sweden | 89.6 | 100.0 | 96.4 | 78.8 | 72.7 | 100 |
| United States | 89.6 | 100.0 | 92.7 | 70.0 | 85.1 | 100 |
| Poland | 89.2 | 100.0 | 96.3 | 71.7 | 78.2 | 100 |
| Slovenia | 89.1 | 100.0 | 95.9 | 79.0 | 70.7 | 100 |
| Ireland | 88.7 | 100.0 | 96.8 | 74.9 | 71.8 | 100 |
| Netherlands | 88.6 | 100.0 | 97.1 | 73.9 | 71.9 | 100 |
| Japan | 88.4 | 100.0 | 89.8 | 76.8 | 80.5 | 95 |
| Korea, Rep. | 88.2 | 100.0 | 92.8 | 79.1 | 83.9 | 85 |
| Mexico | 88.0 | 100.0 | 93.3 | 87.3 | 84.3 | 75 |
| Switzerland | 87.9 | 100.0 | 90.4 | 79.1 | 80.1 | 90 |
| Czech Republic | 87.6 | 100.0 | 89.6 | 77.1 | 76.1 | 95 |
| Germany | 87.5 | 100.0 | 95.3 | 71.6 | 75.4 | 95 |
| Canada | 87.4 | 100.0 | 93.1 | 67.1 | 82.0 | 95 |
| France | 87.2 | 100.0 | 88.8 | 78.4 | 68.9 | 100 |
| Denmark | 87.1 | 90.0 | 97.7 | 70.0 | 77.9 | 100 |
| Spain | 86.8 | 100.0 | 91.2 | 73.2 | 69.4 | 100 |
| Georgia | 86.5 | 100.0 | 91.2 | 85.4 | 70.8 | 85 |
| Lithuania | 86.4 | 100.0 | 92.3 | 70.6 | 69.2 | 100 |
| Slovak Republic | 85.9 | 90.0 | 96.2 | 72.3 | 71.2 | 100 |
| Hungary | 85.8 | 100.0 | 89.0 | 67.4 | 72.4 | 100 |
| Australia | 85.7 | 90.0 | 89.8 | 74.9 | 74.0 | 100 |
| Portugal | 85.6 | 100.0 | 91.9 | 73.6 | 62.7 | 100 |
| Greece | 85.4 | 100.0 | 88.2 | 67.1 | 71.9 | 100 |
| Turkey | 85.3 | 100.0 | 85.6 | 82.8 | 58.2 | 100 |
| Estonia | 84.5 | 90.0 | 91.8 | 70.6 | 70.3 | 100 |
| Romania | 84.5 | 100.0 | 91.1 | 69.3 | 71.9 | 90 |
| Luxembourg | 83.9 | 100.0 | 93.6 | 65.0 | 60.9 | 100 |
| Latvia | 83.7 | 100.0 | 87.9 | 64.8 | 66.0 | 100 |
| United Kingdom | 83.6 | 100.0 | 87.3 | 76.1 | 64.3 | 90 |
| New Zealand | 83.3 | 100.0 | 93.6 | 71.4 | 66.6 | 85 |
| Costa Rica | 83.3 | 100.0 | 87.0 | 86.4 | 73.2 | 70 |
| Iceland | 83.3 | 100.0 | 86.6 | 67.3 | 62.5 | 100 |
| Bulgaria | 83.1 | 100.0 | 91.1 | 63.5 | 70.7 | 90 |
| Albania | 83.0 | 100.0 | 68.6 | 78.3 | 73.0 | 95 |
| Armenia | 82.9 | 100.0 | 84.9 | 83.9 | 60.9 | 85 |
| Chile | 82.6 | 100.0 | 77.7 | 80.7 | 59.8 | 95 |
| Moldova | 82.3 | 100.0 | 95.3 | 63.4 | 62.5 | 90 |
| Cyprus | 82.0 | 100.0 | 91.4 | 59.9 | 73.5 | 85 |
| Belgium | 81.9 | 100.0 | 82.2 | 66.2 | 66.4 | 95 |
| Mongolia | 81.9 | 100.0 | 96.9 | 84.8 | 67.6 | 60 |
| Belarus | 81.3 | 100.0 | 85.3 | 71.8 | 54.5 | 95 |
| Singapore | 81.2 | 100.0 | 99.7 | 54.8 | 71.6 | 80 |
| Serbia | 81.2 | 100.0 | 72.8 | 81.3 | 71.9 | 80 |
| Kazakhstan | 81.0 | 100.0 | 88.3 | 81.2 | 65.5 | 70 |
| Kyrgyz Republic | 80.7 | 100.0 | 81.6 | 83.8 | 53.0 | 85 |
| Russian Federation | 80.2 | 93.4 | 85.8 | 66.6 | 65.1 | 90 |
| Philippines | 79.5 | 100.0 | 93.8 | 78.5 | 85.2 | 40 |
| Thailand | 79.3 | 100.0 | 79.6 | 82.8 | 59.0 | 75 |
| Israel | 78.8 | 100.0 | 86.8 | 64.5 | 47.7 | 95 |
| Brazil | 78.3 | 90.0 | 87.0 | 74.1 | 70.6 | 70 |
| Malta | 78.2 | 100.0 | 83.0 | 53.3 | 74.5 | 80 |
| North Macedonia | 77.7 | 100.0 | 87.9 | 65.6 | 65.2 | 70 |
| South Africa | 77.3 | 90.0 | 86.5 | 83.2 | 62.0 | 65 |
| West Bank and Gaza | 76.9 | 80.0 | 91.8 | 68.1 | 74.4 | 70 |
| Ukraine | 76.8 | 100.0 | 55.1 | 75.2 | 58.9 | 95 |
| Egypt, Arab Rep. | 76.8 | 100.0 | 77.9 | 76.6 | 69.7 | 60 |
| Ecuador | 75.7 | 100.0 | 89.9 | 82.9 | 60.7 | 45 |
| Sri Lanka | 75.5 | 90.0 | 81.2 | 77.6 | 83.8 | 45 |
| Mauritius | 75.1 | 90.0 | 86.7 | 63.9 | 64.8 | 70 |
| Indonesia | 74.9 | 100.0 | 91.7 | 80.0 | 47.8 | 55 |
| Colombia | 74.1 | 100.0 | 85.0 | 85.6 | 44.8 | 55 |
| Croatia | 73.7 | 90.0 | 56.0 | 66.4 | 65.9 | 90 |
| Montenegro | 73.2 | 100.0 | 73.1 | 75.5 | 57.2 | 60 |
| Malaysia | 72.9 | 80.0 | 84.3 | 78.5 | 72.0 | 50 |
| India | 72.7 | 80.0 | 88.3 | 74.9 | 65.0 | 55 |
| Jordan | 72.3 | 80.0 | 82.9 | 81.0 | 62.7 | 55 |
| Saudi Arabia | 71.0 | 80.0 | 81.4 | 55.9 | 77.7 | 60 |
| Azerbaijan | 70.9 | 80.0 | 68.1 | 68.5 | 57.7 | 80 |
| Uruguay | 70.6 | 100.0 | 86.7 | 75.9 | 65.6 | 25 |
| Rwanda | 70.2 | 100.0 | 73.6 | 73.9 | 53.7 | 50 |
| Peru | 69.1 | 90.0 | 86.3 | 84.0 | 50.1 | 35 |
| Vietnam | 68.5 | 100.0 | 68.0 | 60.9 | 78.6 | 35 |
| El Salvador | 68.4 | 90.0 | 79.3 | 68.2 | 44.5 | 60 |
| Senegal | 68.3 | 80.0 | 83.0 | 64.2 | 49.2 | 65 |
| Guatemala | 68.3 | 80.0 | 62.0 | 76.2 | 58.2 | 65 |
| Uganda | 66.9 | 90.0 | 66.6 | 80.9 | 36.8 | 60 |
| United Arab Emirates | 66.5 | 100.0 | 45.3 | 64.7 | 57.5 | 65 |
| Tunisia | 65.9 | 90.0 | 87.3 | 69.6 | 52.8 | 30 |
| Uzbekistan | 65.5 | 60.0 | 74.1 | 69.1 | 44.2 | 80 |
| Myanmar | 65.4 | 100.0 | 67.3 | 81.0 | 43.7 | 35 |
| Panama | 65.3 | 80.0 | 65.9 | 76.5 | 74.0 | 30 |
| Argentina | 65.3 | 70.0 | 80.8 | 81.2 | 59.4 | 35 |
| Bosnia and Herzegovina | 65.0 | 80.0 | 62.7 | 65.0 | 57.5 | 60 |
| Kuwait | 65.0 | 100.0 | 65.2 | 64.3 | 60.5 | 35 |
| Zimbabwe | 65.0 | 90.0 | 66.7 | 75.5 | 42.7 | 50 |
| Paraguay | 64.8 | 70.0 | 61.6 | 82.5 | 45.1 | 65 |
| Bolivia | 64.7 | 100.0 | 66.4 | 71.4 | 55.7 | 30 |
| Dominican Republic | 64.3 | 86.6 | 70.3 | 67.6 | 36.9 | 60 |
| Pakistan | 63.4 | 100.0 | 61.9 | 80.5 | 44.7 | 30 |
| Oman | 63.2 | 100.0 | 47.7 | 54.2 | 68.9 | 45 |
| Morocco | 63.2 | 60.0 | 90.6 | 77.0 | 38.2 | 50 |
| Maldives | 62.9 | 70.0 | 67.7 | 75.8 | 55.9 | 45 |
| Togo | 62.4 | 90.0 | 61.7 | 76.1 | 44.2 | 40 |
| Tanzania | 62.4 | 76.6 | 71.3 | 72.8 | 46.2 | 45 |
| Bangladesh | 61.8 | 90.0 | 60.8 | 79.5 | 53.7 | 25 |
| Cote d'Ivoire | 61.5 | 90.0 | 64.2 | 74.7 | 38.5 | 40 |
| Ghana | 61.3 | 76.6 | 61.5 | 83.0 | 40.4 | 45 |
| Zambia | 61.0 | 90.0 | 67.3 | 74.3 | 33.4 | 40 |
| Brunei Darussalam | 60.9 | 90.0 | 69.3 | 48.4 | 51.6 | 45 |
| Cabo Verde | 60.8 | 80.0 | 64.8 | 68.0 | 71.1 | 20 |
| Qatar | 60.8 | 100.0 | 64.5 | 56.5 | 57.7 | 25 |
| Honduras | 60.5 | 90.0 | 62.2 | 77.1 | 43.4 | 30 |
| Botswana | 60.5 | 60.0 | 67.5 | 70.1 | 64.9 | 40 |
| St. Lucia | 60.2 | 80.0 | 73.6 | 57.8 | 59.9 | 30 |
| Bahrain | 60.0 | 90.0 | 72.2 | 48.3 | 59.6 | 30 |
| Seychelles | 59.5 | 80.0 | 46.2 | 62.0 | 59.3 | 50 |
| Liberia | 59.3 | 90.0 | 62.6 | 70.2 | 33.4 | 40 |
| Belize | 59.0 | 80.0 | 62.4 | 58.3 | 64.3 | 30 |
| Samoa | 58.9 | 90.0 | 67.5 | 69.6 | 27.5 | 40 |
| Malawi | 58.4 | 80.0 | 63.3 | 71.7 | 46.7 | 30 |
| Angola | 58.2 | 80.0 | 62.7 | 69.9 | 43.2 | 35 |
| Cambodia | 58.1 | 70.0 | 61.8 | 72.1 | 41.6 | 45 |
| China | 57.8 | 83.4 | 42.4 | 65.0 | 43.3 | 55 |
| Nepal | 57.3 | 80.0 | 62.7 | 77.0 | 46.8 | 20 |
| Suriname | 57.2 | 60.0 | 69.1 | 60.8 | 61.0 | 35 |
| Lao PDR | 57.0 | 66.6 | 64.3 | 67.6 | 46.5 | 40 |
| Tajikistan | 56.9 | 90.0 | 28.4 | 72.5 | 43.5 | 50 |
| Nigeria | 56.6 | 80.0 | 67.1 | 64.8 | 36.2 | 35 |
| Fiji | 56.6 | 90.0 | 29.2 | 69.0 | 49.8 | 45 |
| Niger | 56.5 | 70.0 | 59.9 | 73.5 | 33.9 | 45 |
| Jamaica | 56.4 | 60.0 | 73.4 | 60.5 | 58.3 | 30 |
| Benin | 56.4 | 70.0 | 69.9 | 74.4 | 32.5 | 35 |
| Tonga | 56.1 | 90.0 | 63.2 | 68.1 | 39.3 | 20 |
| Mozambique | 55.2 | 80.0 | 57.0 | 67.0 | 32.0 | 40 |
| Cameroon | 55.1 | 70.0 | 63.9 | 74.4 | 32.5 | 35 |
| Lebanon | 54.7 | 60.0 | 66.1 | 67.8 | 49.8 | 30 |
| Afghanistan | 54.4 | 80.0 | 59.4 | 72.2 | 20.4 | 40 |
| Sierra Leone | 54.0 | 90.0 | 64.5 | 66.5 | 29.1 | 20 |
| Madagascar | 54.0 | 80.0 | 63.4 | 69.6 | 27.0 | 30 |
| Kenya | 53.8 | 70.0 | 60.7 | 66.2 | 32.2 | 40 |
| Mali | 53.7 | 66.6 | 64.4 | 72.2 | 35.4 | 30 |
| Namibia | 53.6 | 70.0 | 64.7 | 69.5 | 34.0 | 30 |
| Algeria | 53.4 | 80.0 | 25.4 | 71.7 | 49.9 | 40 |
| Burkina Faso | 53.2 | 80.0 | 35.7 | 71.8 | 33.3 | 45 |
| Lesotho | 53.1 | 80.0 | 35.5 | 62.1 | 48.0 | 40 |
| St. Vincent and the Grenadines | 52.8 | 60.0 | 68.5 | 52.4 | 48.3 | 35 |
| Sao Tome and Principe | 52.4 | 60.0 | 61.7 | 58.2 | 52.2 | 30 |
| Nicaragua | 52.3 | 80.0 | 60.3 | 53.8 | 32.5 | 35 |
| Iran, Islamic Rep. | 52.2 | 80.0 | 29.3 | 61.3 | 60.3 | 30 |
| Palau | 52.0 | 70.0 | 57.9 | 47.5 | 44.5 | 40 |
| Guyana | 51.8 | 70.0 | 61.0 | 59.2 | 34.1 | 35 |
| Timor-Leste | 50.9 | 40.0 | 60.6 | 64.2 | 34.8 | 55 |
| Bhutan | 50.9 | 70.0 | 60.6 | 68.3 | 40.5 | 15 |
| Ethiopia | 50.5 | 70.0 | 55.9 | 74.7 | 36.9 | 15 |
| Eswatini | 49.9 | 90.0 | 20.6 | 70.5 | 28.3 | 40 |
| Burundi | 49.7 | 56.6 | 64.0 | 77.0 | 15.7 | 35 |
| Mauritania | 49.5 | 70.0 | 60.9 | 52.7 | 24.0 | 40 |
| Guinea | 49.4 | 80.0 | 61.8 | 62.9 | 22.6 | 20 |
| Bahamas, The | 48.1 | 80.0 | 28.6 | 38.5 | 28.5 | 65 |
| Venezuela, RB | 47.6 | 66.6 | 59.9 | 52.5 | 34.1 | 25 |
| Gambia, The | 47.5 | 60.0 | 32.7 | 78.1 | 36.4 | 30 |
| Congo, Dem. Rep. | 46.2 | 60.0 | 68.1 | 64.6 | 18.2 | 20 |
| Trinidad and Tobago | 45.7 | 60.0 | 27.4 | 58.0 | 43.2 | 40 |
| Papua New Guinea | 44.8 | 60.0 | 57.8 | 65.4 | 15.7 | 25 |
| Dominica | 44.5 | 60.0 | 32.8 | 47.6 | 52.3 | 30 |
| Chad | 44.5 | 70.0 | 57.0 | 62.5 | 18.0 | 15 |
| Antigua and Barbuda | 44.1 | 60.0 | 28.9 | 59.1 | 47.8 | 25 |
| Vanuatu | 42.0 | 56.6 | 59.1 | 57.2 | 21.9 | 15 |
| Iraq | 41.8 | 30.0 | 34.0 | 71.5 | 38.7 | 35 |
| Sudan | 41.5 | 53.4 | 57.9 | 61.6 | 24.6 | 10 |
| Solomon Islands | 41.3 | 50.0 | 59.4 | 57.3 | 14.9 | 25 |
| Djibouti | 40.8 | 50.0 | 59.7 | 51.7 | 17.6 | 25 |
| Haiti | 39.9 | 60.0 | 17.9 | 65.0 | 16.7 | 40 |
| Congo, Rep. | 39.2 | 60.0 | 29.4 | 52.3 | 29.5 | 25 |
| Micronesia, Fed. Sts. | 36.3 | 30.0 | 61.6 | 55.2 | 19.7 | 15 |
| Yemen, Rep. | 35.6 | 66.6 | 27.5 | 47.7 | 16.1 | 20 |
| Marshall Islands | 35.5 | 30.0 | 60.8 | 58.1 | 18.4 | 10 |
| Kiribati | 35.2 | 20.0 | 59.1 | 66.1 | 15.5 | 15 |
| St. Kitts and Nevis | 34.8 | 40.0 | 30.1 | 35.8 | 43.0 | 25 |
| Equatorial Guinea | 33.9 | 10.0 | 59.1 | 52.0 | 23.7 | 25 |
| Guinea-Bissau | 31.9 | 46.6 | 23.7 | 59.6 | 14.6 | 15 |
| Gabon | 30.4 | 40.0 | 25.6 | 50.5 | 16.0 | 20 |
| Somalia | 30.3 | 40.0 | 43.8 | 56.6 | 1.3 | 10 |
| South Sudan | 29.5 | 20.0 | 43.0 | 53.9 | 10.7 | 20 |
| Turkmenistan | 27.2 | 46.6 | 0.5 | 60.5 | 13.3 | 15 |
| Syrian Arab Republic | 25.4 | 16.6 | 25.2 | 43.5 | 16.9 | 25 |
| Libya | 22.9 | 20.0 | 21.3 | 42.9 | 10.3 | 20 |
| American Samoa |  | 40.0 |  | 19.0 |  |  |
| Andorra |  | 80.0 |  | 34.2 |  | 10 |
| Aruba |  | 60.0 |  | 21.3 |  |  |
| Barbados |  | 100.0 |  | 57.4 |  | 35 |
| Bermuda |  | 60.0 |  | 25.0 |  |  |
| British Virgin Islands |  | 60.0 |  | 26.1 |  |  |
| Cayman Islands |  | 70.0 |  | 24.2 |  |  |
| Central African Republic |  | 30.0 |  | 57.2 |  | 20 |
| Channel Islands |  | 60.0 |  |  |  |  |
| Comoros |  | 60.0 |  | 61.2 |  | 30 |
| Cuba |  | 60.0 |  | 67.4 |  |  |
| Curacao |  | 80.0 |  | 26.7 |  |  |
| Eritrea |  | 16.6 |  | 45.1 |  | 15 |
| Faroe Islands |  | 60.0 |  | 15.1 |  |  |
| French Polynesia |  | 60.0 |  | 22.2 |  |  |
| Gibraltar |  | 60.0 |  | 20.1 |  |  |
| Greenland |  | 60.0 |  | 19.4 |  |  |
| Grenada |  | 70.0 |  | 56.0 |  | 20 |
| Guam |  | 60.0 |  | 21.2 |  |  |
| Hong Kong SAR, China |  | 80.0 |  | 31.1 |  |  |
| Isle of Man |  | 70.0 |  | 11.7 |  |  |
| Korea, Dem. People's Rep. |  | 30.0 |  | 50.7 |  |  |
| Kosovo |  | 40.0 | 68.0 |  |  | 60 |
| Liechtenstein |  | 70.0 |  | 31.3 |  |  |
| Macao SAR, China |  | 80.0 |  | 29.4 |  |  |
| Monaco |  | 90.0 |  | 29.6 |  |  |
| Nauru |  | 40.0 |  | 46.4 |  | 5 |
| New Caledonia |  | 70.0 |  | 23.0 |  |  |
| Northern Mariana Islands |  | 60.0 |  | 16.1 |  |  |
| Puerto Rico |  | 80.0 |  | 28.5 |  |  |
| San Marino |  | 90.0 | 58.0 | 28.3 |  | 60 |
| Sint Maarten (Dutch part) |  | 50.0 |  | 16.5 |  |  |
| St. Martin (French part) |  | 40.0 |  | 13.9 |  |  |
| Taiwan, China |  | 60.0 |  |  |  |  |
| Turks and Caicos Islands |  | 60.0 |  | 22.2 |  |  |
| Tuvalu |  | 40.0 |  | 54.8 |  | 5 |
| Virgin Islands (U.S.) |  | 60.0 |  | 18.7 |  |  |

### Supplementary Table A.3: Data Description of SPI_index.csv

| **Name** | **Class** | **Label** | **Values** |
| --- | --- | --- | --- |
| country | character |  |  |
| iso3c | character |  |  |
| date | numeric |  | Num: 2004 to 2020 |
| SPI.INDEX.PIL1 | numeric | Pillar 1 - Data Use – Score | Num: 0 to 100 |
| SPI.INDEX.PIL2 | numeric | Pillar 2 – Data Services – Score | Num: 0.333 to 100 |
| SPI.INDEX.PIL3 | numeric | Pillar 3 – Data Products – Score | Num: 4.894 to 90.938 |
| SPI.INDEX.PIL4 | numeric | Pillar 4 – Data Sources – Score | Num: 0 to 92.6 |
| SPI.INDEX.PIL5 | numeric | Pillar 5 – Data Infrastructure – Score | Num: 0 to 100 |
| SPI.INDEX | numeric | SPI Overall Score | Num: 11.77 to 90.468 |
| SPI.DIM1.5.INDEX | numeric | Dimension 1.5: Data use by international organizations | Num: 0 to 1 |
| SPI.DIM2.1.INDEX | numeric | Dimension 2.1: Data Releases | Num: 0 to 1 |
| SPI.DIM2.2.INDEX | numeric | Dimension 2.2: Online access | Num: 0.004 to 1 |
| SPI.DIM2.4.INDEX | numeric | Dimension 2.4: Data services | Num: 0 to 1 |
| SPI.DIM3.1.INDEX | numeric | Dimension 3.1: Social Statistics | Num: 0 to 0.927 |
| SPI.DIM3.2.INDEX | numeric | Dimension 3.2: Economic Statistics | Num: 0.033 to 0.958 |
| SPI.DIM3.3.INDEX | numeric | Dimension 3.3: Environmental Statistics | Num: 0.084 to 1 |
| SPI.DIM3.4.INDEX | numeric | Dimension 3.4: Institutional Statistics | Num: 0 to 0.945 |
| SPI.DIM4.1.CEN.INDEX | numeric | Dimension 4.1: Censuses | Num: 0 to 1 |
| SPI.DIM4.1.SVY.INDEX | numeric | Dimension 4.1: Surveys | Num: 0 to 1 |
| SPI.DIM4.2.INDEX | Numeric | Dimension 4.2: Administrative Data | Num: 0 to 1 |
| SPI.DIM4.3.INDEX | numeric | Dimension 4.3: Geospatial Data | Num: 0 to 0.795 |
| SPI.DIM5.1.INDEX | numeric | Dimension 5.1: Legislation and governance | Num: -99 to 1 |
| SPI.DIM5.2.INDEX | numeric | Dimension 5.2: Standards and Methods | Num: 0 to 1 |
| SPI.DIM5.5.INDEX | numeric | Dimension 5.5: Finance | Num: -99 to 1 |
| SPI.D1.5.POV | numeric | Dimension 1.5: Data use by international organizations - Availability of Comparable Poverty headcount ratio at $1.90 a day | Num: 0 to 1 |
| SPI.D1.5.CHLD.MORT | numeric | Dimension 1.5: Data use by international organizations - Availability of Mortality rate, under-5 (per 1,000 live births) data meeting quality standards according to UN IGME | Num: 0 to 1 |
| SPI.D1.5.DT.TDS.DPPF.XP.ZS | numeric | Dimension 1.5: Data use by international organizations - Quality of Debt service data according to World Bank | Num: 0 to 1 |
| SPI.D1.5.SAFE.MAN.WATER | numeric | Dimension 1.5: Data use by international organizations - Safely Managed Drinking Water | Num: 0 to 1 |
| SPI.D1.5.LFP | numeric | Dimension 1.5: Data use by international organizations - Labor force participation rate by sex and age (%) | Num: 0 to 1 |
| SPI.D2.1.GDDS | numeric | Dimension 2.1: Data releases - SDDS/e-GDDS subscription | Num: 0 to 1 |
| SPI.D2.2.Machine.readable | numeric | Dimension 2.2: Online access - Machine Readability Score | Num: 0 to 1 |
| SPI.D2.2.Non.proprietary | numeric | Dimension 2.2: Online access - Non-Proprietary format Score | Num: 0 to 1 |
| SPI.D2.2.Download.options | numeric | Dimension 2.2: Online access - Download Options Score | Num: 0 to 1 |
| SPI.D2.2.Metadata.available | numeric | Dimension 2.2: Online access - Metadata Available Score | Num: 0 to 1 |
| SPI.D2.2.Terms.of.use | numeric | Dimension 2.2: Online access - Terms of Use Score | Num: 0 to 1 |
| SPI.D2.2.Openness.subscore | numeric | Dimension 2.2: Online access - ODIN Open Data Openness score | Num: 0.004 to 1 |
| SPI.D2.4.NADA | numeric | Dimension 2.4: Data access services - NADA metadata | Num: 0 to 1 |
| SPI.D3.1.POV | numeric | Dimension 3.1: SDG Goal 1 - GOAL 1: No Poverty | Num: 0 to 1 |
| SPI.D3.2.HNGR | numeric | Dimension 3.2: SDG Goal 2 - GOAL 2: Zero Hunger | Num: 0 to 1 |
| SPI.D3.3.HLTH | numeric | Dimension 3.3: SDG Goal 3 - GOAL 3: Good Health and Well-being | Num: 0 to 1 |
| SPI.D3.4.EDUC | numeric | Dimension 3.4: SDG Goal 4 - GOAL 4: Quality Education | Num: 0 to 0.909 |
| SPI.D3.5.GEND | numeric | Dimension 3.5: SDG Goal 5 - GOAL 5: Gender Equality | Num: 0 to 0.875 |
| SPI.D3.6.WTRS | numeric | Dimension 3.6: SDG Goal 6 - GOAL 6: Clean Water and Sanitation | Num: 0 to 1 |
| SPI.D3.7.ENRG | numeric | Dimension 3.7: SDG Goal 7 - GOAL 7: Affordable and Clean Energy | Num: 0.2 to 1 |
| SPI.D3.8.WORK | numeric | Dimension 3.8: SDG Goal 8 - GOAL 8: Decent Work and Economic Growth | Num: 0 to 0.923 |
| SPI.D3.9.INDY | numeric | Dimension 3.9: SDG Goal 9 - GOAL 9: Industry, Innovation and Infrastructure | Num: 0 to 1 |
| SPI.D3.10.NEQL | numeric | Dimension 3.10: SDG Goal 10 - GOAL 10: Reduced Inequality | Num: 0 to 1 |
| SPI.D3.11.CITY | numeric | Dimension 3.11: SDG Goal 11 - GOAL 11: Sustainable Cities and Communities | Num: 0 to 1 |
| SPI.D3.12.CNSP | numeric | Dimension 3.12: SDG Goal 12 - GOAL 12: Responsible Consumption and Production | Num: 0 to 1 |
| SPI.D3.15.LAND | numeric | Dimension 3.15: SDG Goal 15 - GOAL 15: Life on Land | Num: 0 to 1 |
| SPI.D3.16.INST | numeric | Dimension 3.16: SDG Goal 16 - GOAL 16: Peace and Justice Strong Institutions | Num: 0 to 1 |
| SPI.D3.17.PTNS | numeric | Dimension 3.17: SDG Goal 17 - GOAL 17: Partnerships to achieve the Goal | Num: 0 to 1 |
| SPI.D3.13.CLMT | numeric | Dimension 3.13: SDG Goal 13 - GOAL 13: Climate Action | Num: 0 to 1 |
| SPI.D4.1.1.POPU | numeric | Dimension 4.1: censuses and surveys - Population & Housing census | Num: 0 to 1 |
| SPI.D4.1.2.AGRI | numeric | Dimension 4.1: censuses and surveys - Agriculture census | Num: 0 to 1 |
| SPI.D4.1.3.BIZZ | numeric | Dimension 4.1: censuses and surveys - Business/establishment census | Num: 0 to 1 |
| SPI.D4.1.4.HOUS | numeric | Dimension 4.1: censuses and surveys - Household Survey on income, etc | Num: 0 to 1 |
| SPI.D4.1.5.AGSVY | numeric | Dimension 4.1: censuses and surveys - Agriculture survey | Num: 0 to 1 |
| SPI.D4.1.6.LABR | numeric | Dimension 4.1: censuses and surveys - Labor Force Survey | Num: 0 to 1 |
| SPI.D4.1.7.HLTH | numeric | Dimension 4.1: censuses and surveys - Health/Demographic survey | Num: 0 to 1 |
| SPI.D4.1.8.BZSVY | numeric | Dimension 4.1: censuses and surveys - Business/establishment survey | Num: 0 to 1 |
| SPI.D4.2.3.CRVS | numeric | Dimension 4.2: administrative data - CRVS (WDI) | Num: 0 to 1 |
| SPI.D4.3.GEO.first.admin.level | numeric | Dimension 4.3: geospatial data - Geospatial data available at 1st Admin Level | Num: 0 to 0.795 |
| SPI.D5.1.DILG | numeric | Dimension 5.1: Legislation and governance - Legislation Indicator based on PARIS21 indicators on SDG 17.18.2 | Num: 0 to 1 |
| SPI.D5.2.1.SNAU | numeric | Dimension 5.2: standards - System of national accounts in use | Num: 0 to 1 |
| SPI.D5.2.2.NABY | numeric | Dimension 5.2: standards - National Accounts base year | Num: 0 to 1 |
| SPI.D5.2.3.CNIN | numeric | Dimension 5.2: standards - Classification of national industry | Num: 0 to 1 |
| SPI.D5.2.4.CPIBY | numeric | Dimension 5.2: standards - CPI base year | Num: 0 to 1 |
| SPI.D5.2.5.HOUS | numeric | Dimension 5.2: standards - Classification of household consumption | Num: 0 to 1 |
| SPI.D5.2.6.EMPL | numeric | Dimension 5.2: standards - Classification of status of employment | Num: 0 to 1 |
| SPI.D5.2.7.CGOV | numeric | Dimension 5.2: standards - Central government accounting status | Num: 0 to 1 |
| SPI.D5.2.8.FINA | numeric | Dimension 5.2: standards - Compilation of government finance statistics | Num: 0 to 1 |
| SPI.D5.2.9.MONY | numeric | Dimension 5.2: standards - Compilation of monetary and financial statistics | Num: 0 to 1 |
| SPI.D5.2.10.GSBP | numeric | Dimension 5.2: standards - Business process | Num: 0 to 1 |
| SPI.D5.5.DIFI | numeric | Dimension 5.5: Finance - Finance Indicator based on PARIS21 indicators on SDG 17.18.3 & SDG 17.19.1 | Num: 0 to 1 |
| income | character |  |  |
| region | character |  |  |
| weights | numeric |  | Num: 1 to 1 |
| population | numeric |  | Num: 9828 to 1410929362 |

# Appendix B: Useful Properties of the SPI

We briefly summarize the main technical properties of the SPI and their formal statements below. Based on Atkinson’s (2003) counting method, its weighting structure offers properties such as symmetry, monotonicity, and subgroup decomposability and is. The SPI has a three-level structure, and its overall score is formed by sequentially aggregating the indicators at each level. A full discussion is provided in Cameron *et al.*’s (2021). We use the same notation for better presentation.

A counting method begins with a vector $\boldsymbol{w}=(w_{1},w_{2},\ldots,w_{V})$ containing the values or weights $w_{v}>0$ that will be used to assess the various achievements. The resulting counting index $C$—or the SPI index—is the weighted mean $\mu(\boldsymbol{a};\boldsymbol{w})$ where

$\mu\left( \boldsymbol{a};\boldsymbol{w} \right)$ = $\frac{w_{1}}{w_{1}+\ldots+w_{V}} a_{1}+\ldots+\frac{w_{V}}{w_{1}+\ldots+w_{V}} a_{V}$ (B1)

so that the weight on each $a_{v}$ is $w_{v}/(w_{1}+\ldots+w_{V})$. $C$ takes on values between 0 and 100.

#### Useful Properties

Once $\boldsymbol{w}$ has been set, and the counting index $C$ has been defined, it offers four useful properties. First, $C$ is additively decomposable by subsets of variables or dimensions. For example, define the associated dimensional index $C^{(d)}$ as

$C^{(d)}=\mu(\boldsymbol{a}^{(d)};\boldsymbol{w}^{(d)})=\frac{\boldsymbol{w}^{(d)}{\cdot\boldsymbol{a}}^{(d)}}{\boldsymbol{w}^{(d)}\cdot\boldsymbol{u}^{(d)}}$ (B2)

where $\boldsymbol{w}^{(d)},\boldsymbol{a}^{(d)}$ and $\boldsymbol{u}^{(d)}$ contain only the data associated with variables in dimension *d,* for any $d = 1, 2,\ldots,D$. We can then obtain the overall index *C* as the average of all the dimensional indices

$C= \sum_{d=1}^{D} \frac{1}{D}C^{(d)}$ (B3)

Second, $C$ is additively decomposable by subsets of countries (or regions), which provides a way of monitoring regional or global progress that is based upon the progress in individual countries. Third, $C$ can be constructed using either dichotomous or multi-valued variables. Finally, $C$ allows for different emphasis on progress for countries at different outcome levels.

#### Formal Statements

The proofs for these statements are provided in Cameron *et al.* (2021).

## Proposition 1. Decomposability into Subsets of Variables

*Let* ***S*** *and* ***S****' be two nonempty sets of variables with empty intersection whose union is* $\left\{ a_{1},\ldots,a_{V} \right\}$*. Define the two associated sub-indices of* $C$ *as*

$C^{S}=\mu\left( \boldsymbol{a}^{S};\boldsymbol{w}^{S} \right)=\frac{\boldsymbol{w}^{S}{\cdot\boldsymbol{a}}^{S}}{\boldsymbol{w}^{S}\cdot\boldsymbol{u}^{S}}$ *;* $C^{S'}=\mu\left( \boldsymbol{a}^{S'};\boldsymbol{w}^{S'} \right)=\frac{\boldsymbol{w}^{S'}{\cdot\boldsymbol{a}}^{S'}}{\boldsymbol{w}^{S'}\cdot\boldsymbol{u}^{S'}}$ (B4)

*where* $\boldsymbol{w}^{S},\boldsymbol{a}^{S}$ *and* $\boldsymbol{u}^{\boldsymbol{S}}$ *(or* $\boldsymbol{w}^{S'},\boldsymbol{a}^{S'}$ *and* $\boldsymbol{u}^{S'})$ *are obtained from* $\boldsymbol{w},\boldsymbol{a}$ *and* $\boldsymbol{u}$ *by removing the variables outside of* ***S*** *(respectively,* ***S****').*

*The index C can be decomposed into these two sub-indices as follows*

$C= \frac{\boldsymbol{w}^{S}\cdot\boldsymbol{u}^{S}}{\boldsymbol{w}\cdot\boldsymbol{u}}C^{S}+\frac{\boldsymbol{w}^{S'}\cdot\boldsymbol{u}^{S'}}{\boldsymbol{w}\cdot\boldsymbol{u}}C^{S'}$ (B5)

## Proposition 2. Decomposability into Subsets of Countries

*Let N be the number of countries considered and let* ***A*** *denote the* $N\times V$ *achievement matrix whose n^th^ row* $\boldsymbol{a}^{n}$ *is the achievement vector for country n = 1,…,N.*

*Define the index applied to the collection {1,…,N} of countries as*

$C=C\left( \boldsymbol{A};\boldsymbol{w} \right)=\mu\left( \boldsymbol{A};\boldsymbol{w} \right)$*, where the weighted mean* $\mu\left( \boldsymbol{A};\boldsymbol{w} \right)$ *of matrix* ***A*** *is given by*

$\mu\left( \boldsymbol{A};\boldsymbol{w} \right)=\sum_{n=1}^{N} \sum_{v=1}^{V} (\frac{1}{N} \frac{w_{v}}{w_{1}+\ldots+w_{V}})a_{v}=\sum_{n=1}^{N} \frac{1}{N} \mu(\boldsymbol{a}_{v};\boldsymbol{w})$ (B6)

*The index C can be decomposed into the country-level (or region-level) sub-indices as follows*

$C(\boldsymbol{A};\boldsymbol{w})= \frac{1}{N}\sum_{n=1}^{N} C(\boldsymbol{a}^{n};\boldsymbol{w})$ (B7)

## Proposition 3. Equivalence between Dichotomous and Multi-Valued Indicators

*Suppose that there are T many related dichotomous variables providing information on ascending levels of a component (dimension) of a statistical capacity index. Let* ***S****' be the set of subscripts for these T variables so that* $\boldsymbol{a}^{S'}$ *denotes the relevant vector of achievements. Let* $C\left( \boldsymbol{a}^{S'};\boldsymbol{w}^{S'} \right)$ *be the counting index for this subset of variables; it assigns for* $\boldsymbol{a}^{S'}$ *the T+1 options of the numerical values of 0, 1/T, …, (T-1)/T, and 1, respectively. Now replace the T dichotomous variables with the single variable x having one of these T+1 values and assign it the total weight inherited from the original T variables. The weighted mean across x and the remaining dichotomous variables generates identical index values as the original counting measure.*

# Appendix C: Further Comparison with the SCI

The SPI has several advantages over the SCI on both the conceptual and empirical fronts. Conceptually, it is clearly motivated and offers a framework that is forward looking. The five pillars of data use, data services, data products, data sources, and data infrastructure provide an updated characterization of a modern NSS. It has a number of indicators related to the SDGs that are not available in the SCI. The SPI is also built on a rigorous, axiomatic foundation that satisfies all the desiderata of a statistical capacity index: simple, coherent, motivated, rigorous, implementable, replicable, and incentive consistent (Cameron *et al.,* 2021). Furthermore, the SPI measures more advanced statistical systems in addition to the less mature systems covered in the SCI.

Table C.1 shows that empirically, the SPI offers 51 indicators, which is more than twice the 25 indicators provided by the SCI. While the SCI covers 146 countries and includes no high-income countries, the SPI covers 181 countries including both low-income and high-income countries, which is 24 percent more countries than those of the SCI.^[[1]](#footnote-1)^

As a check on data quality, we calculate the number of unique scores for the SPI overall score. Since tied scores for two (or more) countries does not distinguish these countries’ statistical performance, the fewer tied scores an index has, the better it is. When calculating the number of unique values for the SPI in 2020, we find that there are 181 unique scores for 181 countries (i.e., there are 0 tied values). Put differently, the SPI can distinguish each and every country’s statistical performance in 2020. In contrast, the SCI has only 58 unique values for 145 countries in the same year, implying that it can distinguish only around one-third of these countries regarding their statistical capacity.^[[2]](#footnote-2)^

We next compare the volatility of the SPI and the SCI over time, in terms of the standard deviations of these indices over the five years, 2016 to 2020. The SCI has slightly more volatility during this period with an average standard deviation of 4.2, while the corresponding figure for the SPI is 3.9. For a visual illustration, Figure C.1 plots the relationship between the overall scores in 2016 and 2020 for the two indexes. The SPI (panel on the right) shows a narrower and tighter upward sloping cloud than the SCI (panel on the left) does.

## Supplementary Table C.1: Comparing the SPIs and the SCI

|  | **SCIs** | **SPI** |
| --- | --- | --- |
| Country coverage | 146 | 181+ |
| Time covered | 2004-2019 | 2016 onwards |
| Number of indicators | 25 | 51 |
| Pillars covered | Methodology; Source Data; Periodicity and Timeliness | Data Use, Data Services, Data Products, Data Sources, and Data Infrastructure |
| *Aggregation method* | Simple arithmetic average | Revised weighted average |
| - *Operational relevance* | 1. Track the strengths and weaknesses of country statistical capacity overtime in a cost-effective manner; 2. Track the progress and sustainability of Bank-financed projects in statistical capacity building; 3. With a focus on MDGs in Periodicity section, the old SCIs provided a monitoring tool for country MDGs data production capacity. | 1. Provide an objective, justifiable assessment of country statistical capacity over time with comprehensive, up-to-date information; 2. Provide guidance to the WB teams in assessing the progress and sustainability of the Bank supported projects; 3. Inform Systematic Country Diagnostics; 4. Provide a monitoring tool for countries’ SDGs data production capacity. |
| *Limitations/Identified weaknesses* | 1. 1) Output focused, failing to address the infrastructure/resource part of the NSOs; 2. 2) Relative narrow scope of pillars and indicators; 3. 3) Unable to reflect the change of data landscape and new data requirements brought up by the SDGs. | 1. Selection of indicators under the pillars of data use and data infrastructure are at an early stage of development. 2. In the data sources pillar, indicators on administrative data and geospatial data availability are not comprehensive. 3. Indicators under the data products Pillar are constrained by the development stage of SDGs indicators. |

## Supplementary Figure C.1:  Volatility of SPI and SCI Scores between 2016 and 2020


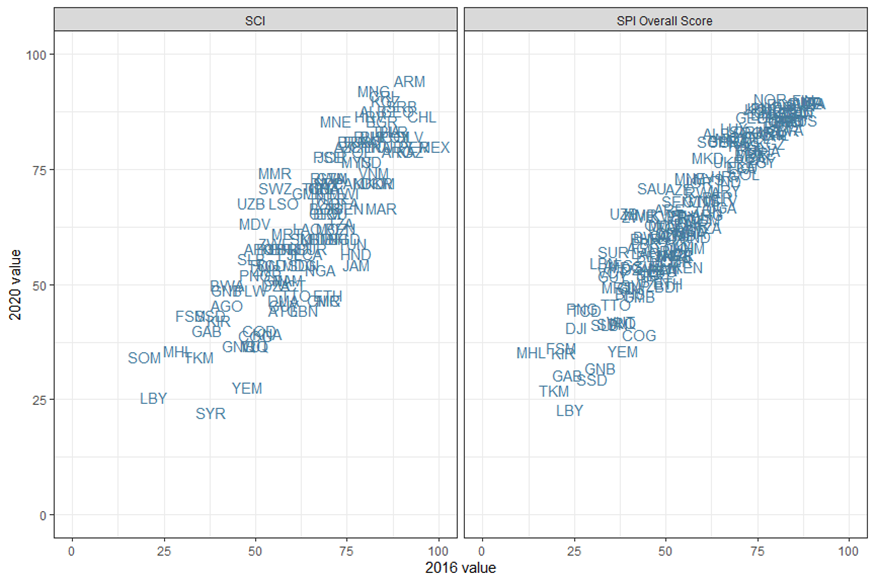


## Code Snippet 1. Expectations for Data Validation

*#define the expectations for each column*

rules <- validator(

countries_included=iso3c %**in**% cntry_list, *#check number of countries included*

dates_covered=date %**in**% c(2004:end_year), *#check dates included*

*# check for each country that the dates are available*

dates_by_country=contains_exactly(

keys=data.frame(date=c(2004:end_year)),

by=iso3c

),

*#go through list of SPI constructed indicators and make sure they are between 0 and 1*

SPI.D1.5.POV_minmax=in_range(SPI.D1.5.POV ,0,1),

SPI.D1.5.CHLD.MORT_minmax=in_range(SPI.D1.5.CHLD.MORT ,0,1),

SPI.D1.5.DT.TDS.DPPF.XP.ZS_minmax=in_range(SPI.D1.5.DT.TDS.DPPF.XP.ZS ,0,1),

SPI.D1.5.SAFE.MAN.WATER_minmax=in_range(SPI.D1.5.SAFE.MAN.WATER ,0,1),

SPI.D1.5.LFP_minmax=in_range(SPI.D1.5.LFP ,0,1),

SPI.D2.1.GDDS_minmax=in_range(SPI.D2.1.GDDS ,0,1),

SPI.D2.2.Machine.readable_minmax=in_range(SPI.D2.2.Machine.readable ,0,1),

SPI.D2.2.Non.proprietary_minmax=in_range(SPI.D2.2.Non.proprietary ,0,1),

SPI.D2.2.Download.options_minmax=in_range(SPI.D2.2.Download.options ,0,1),

SPI.D2.2.Metadata.available_minmax=in_range(SPI.D2.2.Metadata.available ,0,1),

SPI.D2.2.Terms.of.use_minmax=in_range(SPI.D2.2.Terms.of.use ,0,1),

SPI.D2.2.Openness.subscore_minmax=in_range(SPI.D2.2.Openness.subscore ,0,1),

SPI.D2.4.NADA_minmax=in_range(SPI.D2.4.NADA ,0,1),

SPI.D3.1.POV_minmax=in_range(SPI.D3.1.POV ,0,1),

SPI.D3.2.HNGR_minmax=in_range(SPI.D3.2.HNGR ,0,1),

SPI.D3.3.HLTH_minmax=in_range(SPI.D3.3.HLTH ,0,1),

SPI.D3.4.EDUC_minmax=in_range(SPI.D3.4.EDUC ,0,1),

SPI.D3.5.GEND_minmax=in_range(SPI.D3.5.GEND ,0,1),

SPI.D3.6.WTRS_minmax=in_range(SPI.D3.6.WTRS ,0,1),

SPI.D3.7.ENRG_minmax=in_range(SPI.D3.7.ENRG ,0,1),

SPI.D3.8.WORK_minmax=in_range( SPI.D3.8.WORK ,0,1),

SPI.D3.9.INDY_minmax=in_range(SPI.D3.9.INDY ,0,1),

SPI.D3.10.NEQL_minmax=in_range(SPI.D3.10.NEQL ,0,1),

SPI.D3.11.CITY_minmax=in_range(SPI.D3.11.CITY ,0,1),

SPI.D3.12.CNSP_minmax=in_range(SPI.D3.12.CNSP ,0,1),

SPI.D3.15.LAND_minmax=in_range(SPI.D3.15.LAND ,0,1),

SPI.D3.16.INST_minmax=in_range(SPI.D3.16.INST ,0,1),

SPI.D3.17.PTNS_minmax=in_range(SPI.D3.17.PTNS ,0,1),

SPI.D3.13.CLMT_minmax=in_range(SPI.D3.13.CLMT ,0,1),

SPI.D4.1.1.POPU_minmax=in_range(SPI.D4.1.1.POPU ,0,1),

SPI.D4.1.2.AGRI_minmax=in_range(SPI.D4.1.2.AGRI ,0,1),

SPI.D4.1.3.BIZZ_minmax=in_range(SPI.D4.1.3.BIZZ ,0,1),

SPI.D4.1.4.HOUS_minmax=in_range(SPI.D4.1.4.HOUS ,0,1),

SPI.D4.1.5.AGSVY_minmax=in_range(SPI.D4.1.5.AGSVY ,0,1),

SPI.D4.1.6.LABR_minmax=in_range(SPI.D4.1.6.LABR ,0,1),

SPI.D4.1.7.HLTH_minmax=in_range(SPI.D4.1.7.HLTH ,0,1),

SPI.D4.1.8.BZSVY_minmax=in_range(SPI.D4.1.8.BZSVY ,0,1),

SPI.D4.2.3.CRVS_minmax=in_range(SPI.D4.2.3.CRVS ,0,1),

SPI.D4.3.GEO.first.admin.level_minmax=in_range(SPI.D4.3.GEO.first.admin.level ,0,1),

SPI.D4.3.GEO.second.admin.level_minmax=in_range(SPI.D4.3.GEO.second.admin.level ,0,1),

SPI.D5.1.DILG_minmax=in_range(SPI.D5.1.DILG ,0,1),

SPI.D5.2.1.SNAU_minmax=in_range(SPI.D5.2.1.SNAU ,0,1),

SPI.D5.2.2.NABY_minmax=in_range(SPI.D5.2.2.NABY ,0,1),

SPI.D5.2.3.CNIN_minmax=in_range(SPI.D5.2.3.CNIN ,0,1),

SPI.D5.2.4.CPIBY_minmax=in_range(SPI.D5.2.4.CPIBY ,0,1),

SPI.D5.2.5.HOUS_minmax=in_range(SPI.D5.2.5.HOUS ,0,1),

SPI.D5.2.6.EMPL_minmax=in_range(SPI.D5.2.6.EMPL ,0,1),

SPI.D5.2.7.CGOV_minmax=in_range(SPI.D5.2.7.CGOV ,0,1),

SPI.D5.2.8.FINA_minmax=in_range(SPI.D5.2.8.FINA ,0,1),

SPI.D5.2.9.MONY_minmax=in_range(SPI.D5.2.9.MONY ,0,1),

SPI.D5.2.10.GSBP_minmax=in_range(SPI.D5.2.10.GSBP ,0,1),

SPI.D5.3.DISK_minmax=in_range(SPI.D5.3.DISK ,0,1),

SPI.D5.5.DIFI_minmax=in_range(SPI.D5.5.DIFI ,0,1)

)

out <- confront(SPI_df_new, rules)

summary(out)

1. Of these 51 indicators, 44 indicators are used to construct the SPI overall score. [↑](#footnote-ref-1)
2. For each specific pillar of the SPI, there are 18 unique scores for Pillar 1 on data use. The data use indicator is coming solely from pillar 1.5 on data use by international organizations. For Pillar 2, there are 163 unique scores, whereas for Pillars 3 and 4 there are 172 unique scores. Lastly, there are 20 unique scores for pillar 5. [↑](#footnote-ref-2)
